# High-fat diet-induced L-saccharopine accumulation inhibits estradiol synthesis and damages oocyte quality by disturbing mitochondrial homeostasis

**DOI:** 10.1080/19490976.2024.2412381

**Published:** 2024-10-16

**Authors:** Jingyi Wen, Yanzhi Feng, Liru Xue, Suzhen Yuan, Qian Chen, Aiyue Luo, Shixuan Wang, Jinjin Zhang

**Affiliations:** a Department of Obstetrics and Gynecology, Tongji Hospital, Tongji Medical College, Huazhong University of Science and Technology, Wuhan, Hubei, China; b National Clinical Research Center for Obstetrical and Gynecological Diseases, Tongji Hospital, Tongji Medical College, Huazhong University of Science and Technology, Wuhan, Hubei, China; c Key Laboratory of Cancer Invasion and Metastasis, Ministry of Education, Wuhan, Hubei, China

**Keywords:** Infertility, ovarian steroidogenesis, oocyte quality, high-fat diet, gut microbiota, L-saccharopine, mitochondrial homeostasis

## Abstract

High-fat diet (HFD) has been linked to female infertility. However, the specific age at which HFD impacts ovarian function and the underlying mechanisms remain poorly understood. Here, we administered a HFD to female mice at various developmental stages: pre-puberty (4 weeks old), post-puberty (6 weeks old), young adult (9 weeks old), and middle age (32 weeks old). Our observations indicated that ovarian function was most significantly compromised when HFD was initiated at post-puberty. Consequently, post-puberty mice were chosen for further investigation. Through transplantation of fecal bacteria from the HFD mice to the mice on a normal diet, we confirmed that gut microbiota dysbiosis contributed to HFD-induced deteriorated fertility and disrupted estradiol synthesis. Utilizing untargeted and targeted metabolomics analyses, we identified L-saccharopine as a key metabolite, which was enriched in the feces, serum, and ovaries of HFD and HFD-FMT mice. Subsequent *in vitro* and *in vivo* experiments demonstrated that L-saccharopine disrupted mitochondrial homeostasis by impeding AMPKα/MFF-mediated mitochondrial fission. This disruption ultimately hindered estradiol synthesis and compromised oocyte quality. AICAR, an activator of AMPKα, ameliorated L-saccharopine induced mitochondrial damage in granulosa cells and oocytes, thereby enhancing E2 synthesis and improving oocyte quality. Collectively, our findings indicate that the accumulation of L-saccharopine may play a pivotal role in mediating HFD-induced ovarian dysfunction. This highlights the potential therapeutic benefits of targeting the gut microbiota-metabolite-ovary axis to address HFD-induced ovarian dysfunction.

## Graphical abstract

**Figure:**
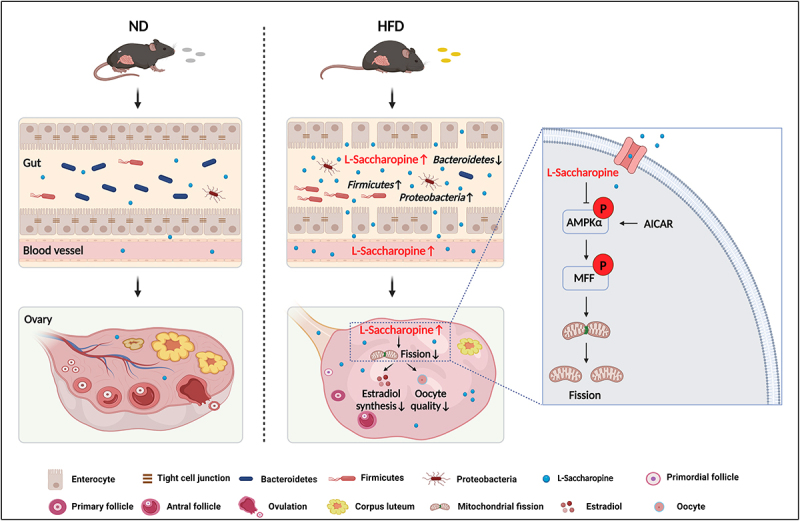
HFD induced gut microbiota dysbiosis and altered metabolome profile, which impaired the intestinal mucosal barrier and led to accumulation of L-saccharopine, a metabolite enriched in the feces, serum, and ovaries of HFD mice. The increased L-saccharopine content disrupted mitochondrial homeostasis by inhibiting AMPKα/MFF-mediated mitochondrial fission, thereby inhibiting estradiol synthesis and damaging oocyte quality (image created with BioRender.com). ND, normal diet; HFD, high-fat diet; AICAR, acadesine.

## Introduction

Ovarian function is essential for female fertility and overall health,^1^ with disruptions leading to negative consequences on individual well-being, personal autonomy, and familial contentment.^2^ Various factors, including genetics, environmental conditions, and dietary habits, can impact ovarian function.^3^ High-fat diet (HFD) presents a significant public health concern, as it is linked to the development of obesity and has been correlated with various health issues such as heart disease,^4–6^ neuroinflammation,^7^ tumorigenesis,^8^ and infertility.^9–11^ Animal studies have shown that HFD can result in dysfunction of the hypothalamic-pituitary-ovarian axis, ovulatory disorders, impaired steroid hormone synthesis, and disrupted follicular development, ultimately leading to ovarian damage and reduced fertility.^12–15^ These effects may be correlated with inflammation, endoplasmic reticulum stress, and impaired ovarian DNA damage repair.^16–18^ However, the underlying molecular mechanisms remain incompletely understood.

The human gastrointestinal tract hosts a dynamic community of approximately 100 trillion symbiotic microbes,^19^ which can be influenced by specific foods and dietary patterns.^20^ HFD-induced gut microbiota dysbiosis is characterized by a decrease in the total microbial community, variations in bacterial species abundance (most notably, an increased proportion of individuals belonging to the phylum *Firmicutes*, and a decreased proportion of individuals belonging to the phylum *Bacteroidetes*), and a concomitant increase in intestinal permeability.^21–23^ This dysbiosis has been linked to several diseases, including male infertility, Alzheimer’s disease (AD), and fatty liver.^10,24,25^ Although recent studies have identified a connection between ovarian function and gut microbiota,^26,27^ it remains uncertain whether dysbiosis of the gut microbiota contributes to HFD-induced ovarian dysfunction.

To address this question, female mice at various developmental stages, including pre-puberty (4 weeks old), post-puberty (6 weeks old), young adult (9 weeks old), and middle age (32 weeks old), were subjected to a HFD regimen. The impact of the HFD on ovarian function was subsequently assessed within each age group. To further explore the role of gut microbiota dysbiosis in HFD-induced ovarian damage, a fecal microbiota transplantation (FMT) study was conducted. Additionally, we utilized metagenomics, metabolomics, and transcriptomics techniques to identify critical metabolites and elucidate the underlying mechanisms through which HFD-induced gut microbiota dysbiosis disrupts ovarian function, which will provide potential therapeutic targets to restore ovarian function.

## Results

### Effects of HFD on ovarian function depended on the age at intervention

**Figure 1.**
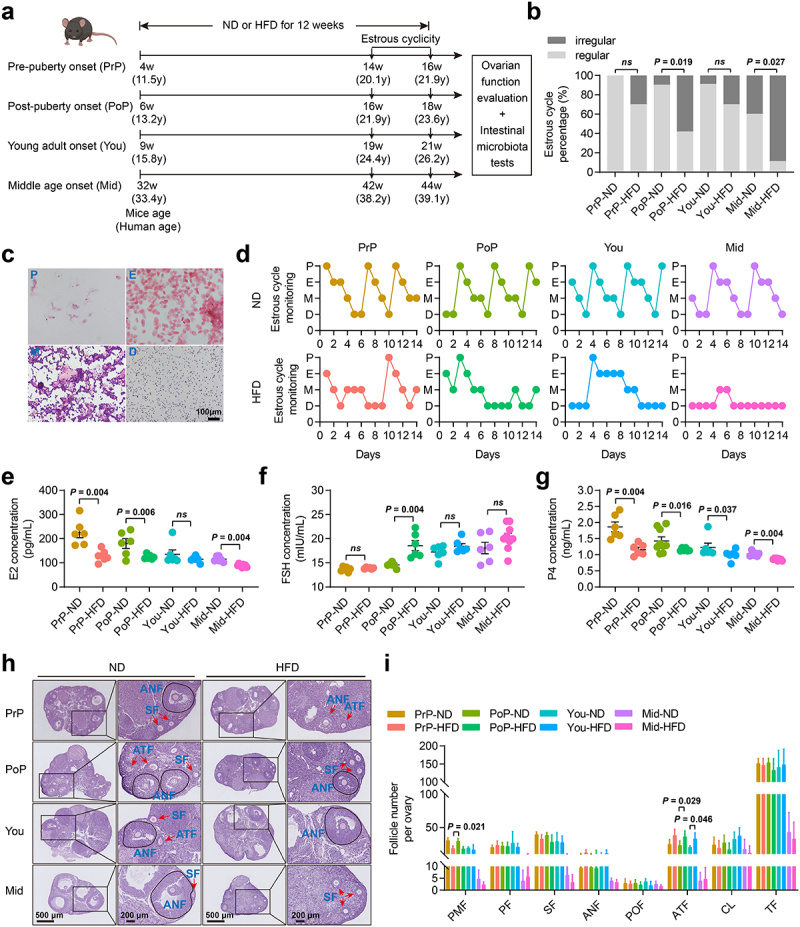
HFD induced ovarian dysfunction at different ages. a, Schematic representation of HFD intervention strategy in mice of different age groups. b, Percentage of irregular estrous cycles (*n* ≥ 9). c, Representative H&E staining images of the estrous cycle. Scar bar, 100 μm. P, proestrus; E, estrus; M, metestrus; D, diestrus. d, Representative estrous cycles. e-g, Serum E2 (e), FSH (f), and P4 (g) levels in mice (*n* = 6). h, Representative H&E staining images of mice ovaries. Scale bars, 500 μm and 200 μm. i, Follicle counts for ovarian serial sections (*n* = 3 - 4). Individual values are displayed as dots, while mean ± SEM is shown as a column and error bar. Statistical significance was determined by Chi-square test (b), or Kruskal – Wallis rank sum test (e, f, g, i). *p* < 0.05 was considered statistically significant. ND, normal diet; HFD, high-fat diet; PrP, pre-puberty; PoP, post-puberty; you, young adult; mid, middle age; E2, estradiol; FSH, follicle-stimulating hormone; P4, progesterone; *ns*, not significant; PMF, primordial follicle; PF, primary follicle; SF, secondary follicle; ANF, antral follicle; POF, pre-ovulatory follicle; ATF, atretic follicle; CL, corpus luteum; TF, total follicle.

To determine if the timing of dietary intervention affects ovarian function, female mice at different developmental stages – pre-puberty, post-puberty, young adult, and middle age – were administered either a HFD or a normal diet (ND) (Figure 1a). All comparisons between the ND- and HFD-treated mice were made within the same age group. After 12 weeks of dietary intervention, the body weights of the HFD mice increased significantly (Fig. S1a-h). Impaired glucose tolerance was observed in all HFD groups, except for the pre-puberty mice, as illustrated in Fig. S1i-p. The serum levels of estradiol (E2) and progesterone (P4) were notably reduced in mice that began the HFD regimen during pre-puberty (PrP-HFD). In mice that initiated the HFD during young adulthood (You-HFD), we observed a significant decrease in serum P4 concentrations and an increase in the number of atretic follicles. Mice that started the HFD at middle age (Mid-HFD) showed a significant increase in the proportion of irregular estrous cycles, accompanied by a notable decrease in serum E2 and P4 levels. Notably, only the mice beginning the HFD at post-puberty (PoP-HFD) demonstrated significant alterations in the serum concentrations of E2, P4, and FSH concurrently, with marked decreases in E2 and P4 and a significant increase in FSH. Additionally, the PoP-HFD group exhibited a higher prevalence of irregular estrous cycles and a significant reduction in the number of primordial follicles, and a notable increase in atretic follicles (Figure 1b-i). Therefore, these findings indicated that a HFD may have detrimental effects on ovarian function in mice of varying ages, particularly in individuals who begin the HFD in post-puberty.

### HFD caused gut microbiota dysbiosis

**Figure 2.**
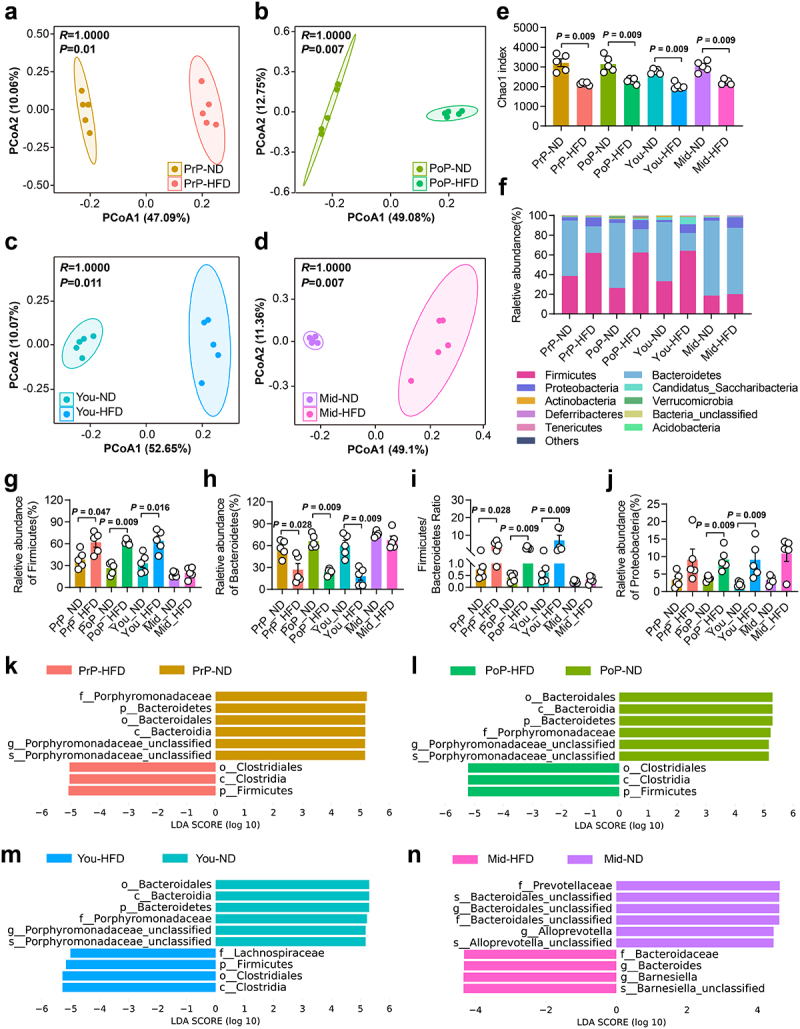
HFD induced gut microbiota dysbiosis at different ages. a-d, PCoA plots of gut microbiota based on the operational unit metrics of samples from ND- and HFD-treated pre-puberty (a), post-puberty (b), young adult (c), and middle age (d) mice, respectively (*n* = 5). e, Chao1 index of the gut microbiota in mice (*n* = 5). f, Relative abundance of microbiota at the phylum level in the indicated groups (*n* = 5). g, Comparison of the relative abundance of *Firmicutes* (*n* = 5). h, Comparison of the relative abundance of *bacteroidetes* (*n* = 5). i, Comparison of the *Firmicutes*/*Bacteroidetes* ratio (*n* = 5). j, Comparison of the relative abundance of *proteobacteria* (*n* = 5). k-n, LDA scores (log 10) analyzed by LEfSe showing bacterial taxa with significantly different abundances between mice treated with ND and HFD from pre-puberty (k), post-puberty (l), young adult (m), and middle age (n), respectively (*n* = 5). Individual values are displayed as dots, while mean ± SEM is shown as a column and error bar. Statistical significance was determined by Kruskal – Wallis rank sum test (e, g-j). *p* < 0.05 was considered statistically significant. ND, normal diet; HFD, high-fat diet; PrP, pre-puberty; PoP, post-puberty; you, young adult; mid, middle age; PCoA, principal coordinate analysis; LEfSe, linear discriminant analysis effect size; LDA, linear discriminant analysis.

We further investigated the impact of HFD on the structure of gut microbiota community using 16S rRNA gene sequencing. Unweighted principal co-ordinates analysis (PCoA) clearly showed segregation in bacterial composition between the HFD and ND mice across all age groups (Figure 2a-d). Consistent with previous reports,^21^ the richness of gut microbiota was significantly reduced in HFD-treated mice across all age groups (Figure 2e). Additionally, apart from the middle-aged group, the HFD significantly increased the *Firmicutes*/*Bacteroidetes* ratio in the other three age groups, compared with that in the ND mice. The HFD also significantly increased *Proteobacteria* abundance in post-puberty and young adult mice (Figure 2f-j). Linear discriminant analysis effect size (LEfSe) analyses revealed consistent patterns of gut microbiota changes in pre-puberty, post-puberty, and young adult mice following HFD intervention (Figure 2k-n). These findings suggested that a HFD disturbed gut microbiota community across all age groups. Given that initiating a HFD in post-puberty had the most significant impact on ovarian function, we chose the post-puberty group for further study.

### Hfd-induced gut microbiota dysbiosis deteriorated fertility and disrupted ovarian steroidogenesis

**Figure 3.**
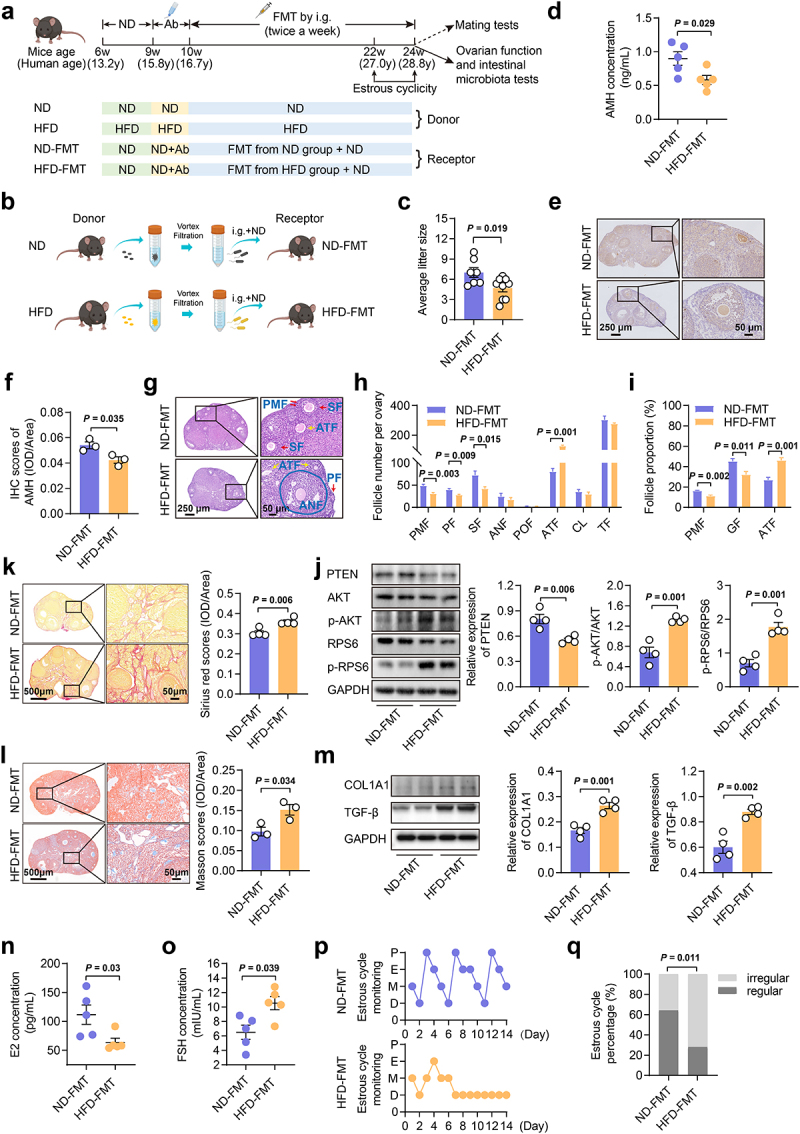
Fecal microbiota transplanted from the HFD mice damaged ovarian function in recipient mice. a-b, Schematic diagram of the FMT experiment. c, Average litter size for each female mouse (*n* ≥ 7). d, Serum AMH levels in mice (*n* = 5). e, f, Protein expression of AMH in the ovary of mice by immunohistochemistry (*n* = 3). Scale bars, 250 μm and 50 μm. g, Representative H&E staining images of mouse ovaries. Scale bars, 250 μm and 50 μm. h, Follicle counting results according to ovarian serial sections (*n* = 5). i, Proportion of follicles at different stages (*n* = 5). j, Protein expression of PTEN, p-AKT, AKT, p-RPS6, and RPS6 in the ovary of mice by western blot (*n* = 4). k, Representative images for Sirius Red staining of ovaries and Sirius Red staining scores of ovaries based on IOD/Area (*n* = 4). Scale bars, 500 μm and 50 μm. l, Representative images for Masson staining of ovaries and Masson staining scores of ovaries based on IOD/Area (*n* = 3). Scale bars, 500 μm and 50 μm. m, Protein expression of COL1A1 and TGF-β in the ovary of mice by western blotting (*n* = 4). n, Serum E2 concentrations in mice (*n* = 5). o, Serum FSH concentrations in mice (*n* = 5). p, Representative estrous cycles. P, proestrus; E, estrus; M, metestrus; D, diestrus. q, Percentage of irregular estrous cycles (*n* = 25). Individual values are displayed as dots, while the mean ± SEM is shown as a column and error bar. Statistical significance was determined by Chi-square test (q), or a two-tailed unpaired Student’s *t*-test. *p* < 0.05 was considered statistically significant. ND, normal diet; HFD, high-fat diet; Ab, antibiotics; FMT, fecal microbiota transplantation; AMH, anti-Müllerian hormone; PMF, primordial follicle; PF, primary follicle; SF, secondary follicle; ANF, antral follicle; POF, pre-ovulatory follicle; ATF, atretic follicle; CL, corpus luteum; TF, total follicle; GF, growing follicle; E2, estradiol; FSH, follicle-stimulating hormone.

To explore whether HFD-induced gut microbiota dysbiosis could damage ovarian function, fecal bacteria from the HFD and ND donors were transplanted into female mice maintained on the ND. The FMT procedures were conducted twice weekly over 14 weeks (Figure 3a,b). Following the microbial transplantation regimen, the donor HFD mice exhibited comparable alterations in obesity phenotype, ovarian function, and gut microbiota similar to those observed in the PoP-HFD mice in the aforementioned experiment (Fig. S2-S4). Then, we evaluated the effects of FMT on body weight, lipid levels, glucose tolerance, insulin resistance, and ovarian function in mice. There were no significant differences in metabolic parameters between mice that received gut microbes from the ND donors (ND-FMT) and those that received gut microbes from the HFD donors (HFD-FMT) (Fig. S5). Notably, HFD-FMT resulted in reduced fertility, as indicated by a significant reduction in average litter size (Figure 3c). Additionally, we found significantly decreased levels of anti-Müllerian hormone (AMH), an indicator of ovarian reserve,^28^ in both serum and ovarian tissues (Figure 3d-f). This was accompanied by elevated levels of FSH in the serum, a reduction in primordial follicles, and an increase in atretic follicles (Figures 3g-i, o), indicating diminished ovarian reserve in the HFD-FMT mice. HFD-FMT remarkably downregulated the expression of PTEN, which is an identified inhibitory factor in primordial follicle activation.^29^ Meanwhile, the AKT-RPS6 signaling pathway, a classic pathway of primordial follicle activation,^29^ was activated in the same mice (Figure 3j). These results suggested that HFD-induced gut microbiota dysbiosis promoted primordial follicle activation, which may be one of the important causes of diminished ovarian reserve.^30^ A recent study showed that ovarian fibrosis may contribute to impaired fertility in mice, especially in obese mice.^31^ Sirius red staining, Masson staining, and fibrosis marker (COL1A1 and TGF-β^32^) expression all indicated an increased degree of ovarian fibrosis in the HFD-FMT mice (Figure 3k-m). Besides, the decrease of serum E2 levels and disturbance of estrous cycles both suggested ovarian endocrine dysfunction in the HFD-FMT mice (Figure 3n,p,). Taken together, these findings indicated that HFD-induced gut microbiota dysbiosis could result in ovarian dysfunction without inducing significant metabolic dysregulations.

**Figure 4.**
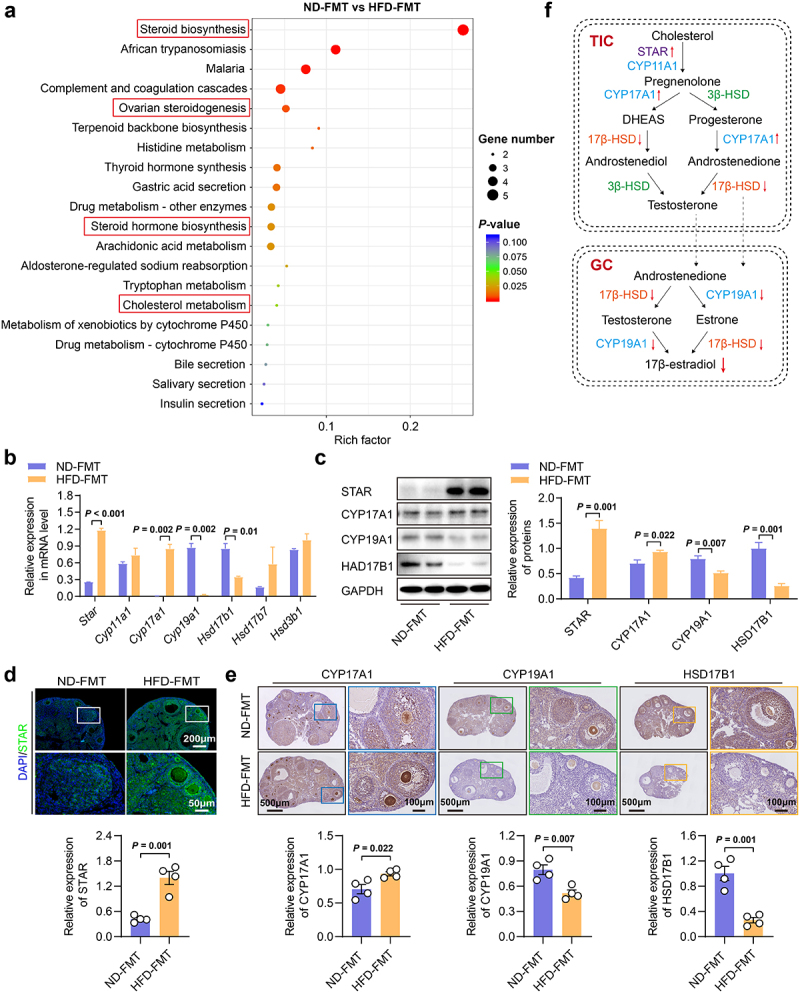
Fecal microbiota transplanted from the HFD mice disrupted ovarian steroidogenesis in recipient mice. a, KEGG enrichment of differentially expressed genes from RNA-Seq of ovarian tissues for ND-FMT and HFD-FMT mice (*n* = 5). b, mRNA expression of *Star*, *Cyp11a1*, *Cyp17a1*, *Cyp19a1*, *Hsd17b1*, *Hsd17b7*, and *Hsd3b1* in the ovary of mice by qRT-PCR (*n* = 4). c, Protein expression of STAR, CYP17A1, CYP19A1, and HSD17B1 in the ovary of mice by western blotting (*n* = 4). d, Protein expression of STAR in the ovary of mice by immunofluorescence. Scale bars, 200 μm and 50 μm. e, Protein expression of CYP17A1, CYP19A1, and HSD17B1 in the ovary of mice by immunohistochemistry (*n* = 3). Scale bars, 500 μm and 100 μm. f, Diagram of molecules in the ovarian steroidogenesis pathway. Individual values are displayed as dots, while the mean ± SEM is shown as a column and error bar. Statistical significance was determined by a two-tailed unpaired Student’s *t*-test. *p* < 0.05 was considered statistically significant. ND, normal diet; HFD, high-fat diet; FMT, fecal microbiota transplantation; TIC, theca-interstitial cell; GC, granulosa cell.

To gain insights into the mechanisms of ovarian dysfunction, we performed RNA sequencing on the ovaries of FMT mice. A total of 179 differentially expressed genes (DEGs) were identified between the ND-FMT and HFD-FMT groups (Fig. S6). Kyoto Encyclopedia of Genes and Genomes (KEGG) enrichment analysis showed that DEGs were significantly enriched in multiple metabolic pathways related to ovarian steroid hormone synthesis, including steroid biosynthesis, ovarian steroidogenesis, steroid hormone biosynthesis, and cholesterol metabolism (Figure 4a). Considering that steroid hormones are required for the successful establishment and maintenance of pregnancy and proper development of the embryo and fetus,^33^ we next examined the expression of genes encoding rate-limiting enzymes in the steroidogenesis pathway at the mRNA and protein levels.^34^ Significantly, we observed that the expression levels of STAR and CYP17A1 were substantially higher in the HFD-FMT group compared to the ND-FMT group, whereas the expression levels of CYP19A1 and HSD17B1 were markedly reduced (Figure 4b-e). CYP19A1 and HSD17B1 are rate-limiting enzymes essential for E2 synthesis in ovarian granulosa cells, with CYP19A1 facilitating the conversion of testosterone to E2 and HSD17B1 converting estrone to E2.^34^ Therefore, we speculated that HFD-induced gut microbiota dysbiosis disturbed ovarian function primarily by disrupting ovarian steroidogenesis, especially E2 synthesis in granulosa cells (Figure 4f). However, the mechanism by which gut microbiota communicate with the ovary remains unclear.

### HFD-induced gut microbiota dysbiosis disrupted the intestinal mucosal barrier

**Figure 5.**
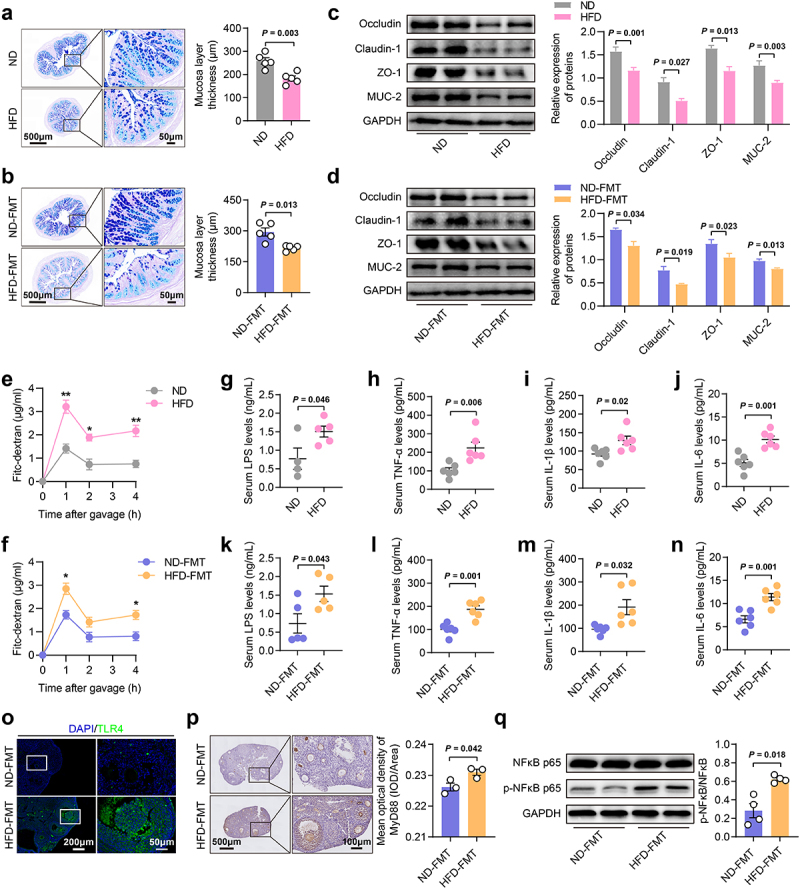
HFD-induced gut microbiota dysbiosis disrupted the intestinal mucosal barrier. a, b, Representative histological images of intestinal mucosal layer and thickness of the mucosa layer in mice (*n* = 5). Scale bars, 500 μm and 50 μm. c, d, Protein expression of Occludin, claudin-1, ZO-1, and MUC-2 in the colon of mice by western blotting (*n* = 4). e, f, Serum levels of FITC-dextran after an oral load in mice (*n* = 3). g-n, Serum LPS, TNF-α, IL-1β, and IL-6 levels in mice (*n* = 4-5). o, Protein expression of TLR4 in the ovary of mice by immunofluorescence. Scale bars, 200 μm and 50 μm. p, Protein expression of MYD88 in the ovary of mice by immunohistochemistry (*n* = 3). Scale bars, 500 μm and 100 μm. q, Protein expression of NFκB and p- NFκB in the ovary of mice by western blotting (n = 4). Individual values are displayed as dots, while the mean ± SEM is shown as a column and error bar. Statistical significance was determined by a two-tailed unpaired Student’s *t*-test. *p* < 0.05 was considered statistically significant. *p < 0.05, **p < 0.01. ND, normal diet; HFD, high-fat diet; FMT, fecal microbiota transplantation; LPS, lipopolysaccharide; TNF, tumor necrosis factor; IL, interleukin.

The intestinal mucosal barrier serves as a crucial defense, protecting the host from various ingested toxins and microbes. In our study, we observed a disruption of this barrier in the colon of HFD and HFD-FMT mice, as indicated by thinning of the colonic mucosa (Figure 5a,b) and decreased expression of barrier proteins (Figure 5c,d), aligning with findings from previous studies.^22^ Further analysis of intestinal permeability, following the oral administration of FITC-coupled dextran, revealed a notable increase in permeability in both HFD and HFD-FMT mice (Figure 5e,f). Damage to the intestinal mucosal barrier may facilitate the translocation of lipopolysaccharides (LPS) into the systemic circulation, which can activate downstream signaling pathways and impact extraintestinal organs.^35^ In this study, we also detected significantly increased serum levels of LPS and barrier-disrupting cytokines such as tumor necrosis factor (TNF)-α, interleukin (IL)-1β, and IL-6^22^ in the HFD and HFD-FMT mice (Figure 5g-n). Additionally, activation of the TLR4/MYD88/NF-κB signaling pathway was also observed in the ovaries of HFD-FMT mice (Figure 5o-q).

### L-saccharopine was enriched in the feces, serum, and ovaries of HFD and HFD-FMT mice

**Figure 6.**
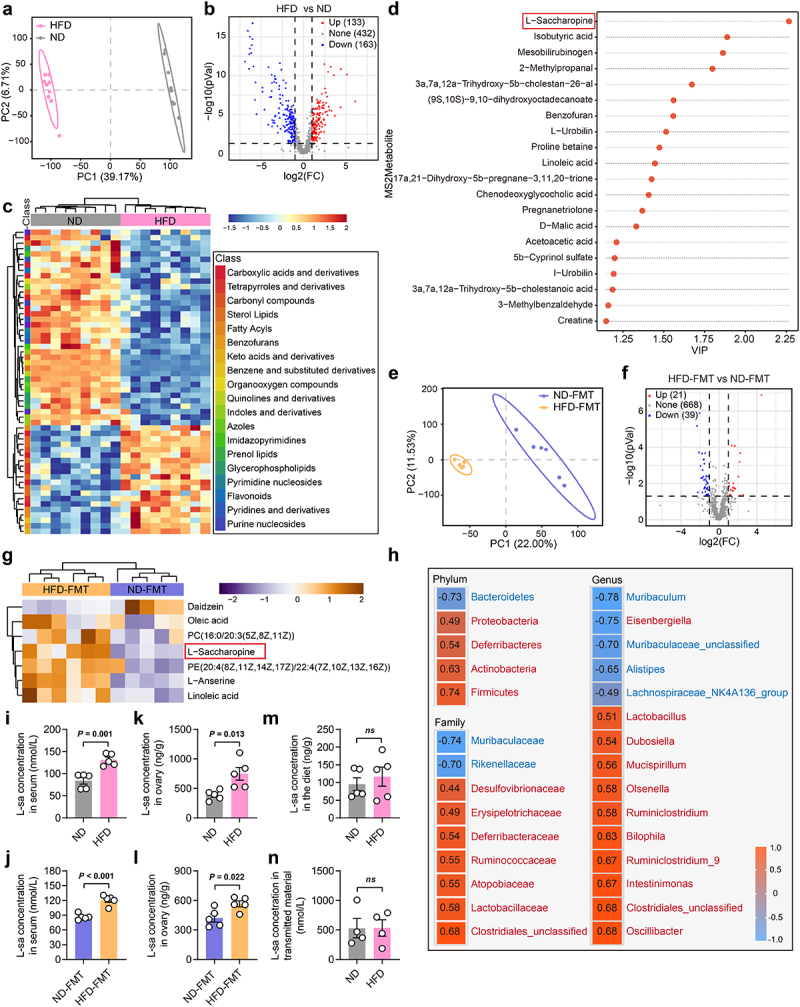
HFD and HFD-induced gut microbiota dysbiosis remodeled the metabolome pattern of gut microbes. a, PLS-DA showing differential clustering of metabolites in feces among the ND and HFD mice (*n* = 9). b, Volcano plot of metabolites. Red and blue dots indicate the up-regulated and down-regulated metabolites in the HFD group, respectively. c, Heatmaps of differential metabolites between the ND and HFD group. d, VIP scores of the 20 upregulated metabolites in the HFD group that could be matched to functional annotations in the KEGG database. e, Fecal metabolic profile was significantly different between the ND-FMT and HFD-FMT group, based on PLS-DA method (*n* = 6 for the ND-FMT group, *n* = 5 for the HFD-FMT group). f, Volcano plot of metabolites. Red and blue dots indicate the upregulated and downregulated metabolites in the HFD-FMT group, respectively. g, Heatmaps of differential metabolites between the ND-FMT and HFD-FMT group. h, Spearman correlation between significantly abundant bacteria (relative abundance > 1%) and fecal L-saccharopine content among the ND and HFD mice. Numbers in the figure indicate Spearman’s correlation coefficients. Bacteria with names in red font were significantly increased in the HFD group, while those in blue were significantly decreased in the HFD group. i-l, Serum and ovarian L-saccharopine levels of the ND, HFD, ND-FMT, and HFD-FMT mice measured by UHPLC-MS/MS (*n* = 5). m, n, Concentration analysis of L-saccharopine in the diet (*n* = 5) and transmitted material (*n* = 4) from the ND and HFD groups. Individual values are displayed as dots, while mean ± SEM is shown as a column and error bar. Statistical significance was determined using a two-tailed unpaired Student’s *t*-test (i-n). *p* < 0.05 was considered statistically significant. ND, normal diet; HFD, high-fat diet; FMT, fecal microbiota transplantation; PLS-DA, partial least squares discriminant analysis; FC, fold change; VIP, variable importance in project; *ns*, no significant difference; L-Sa, L-saccharopine.

To further explore the key molecules involved in the gut microbiota-ovary axis, we performed untargeted metabolomics profiling of fecal samples using ultra-high performance liquid chromatography-mass spectrometry (UHPLC-MS). Partial least squares discriminant analysis (PLS-DA) revealed distinct clustering of metabolites between the ND and HFD groups (Figure 6a). The HFD triggered widespread changes in metabolites (Figure 6b,c). Among those, 20 upregulated metabolites could be matched to functional annotations in the KEGG database. The variable importance in projection (VIP) score highlighted that L-saccharopine contributed most significantly to the group clustering (Figure 6d). Significant differences in metabolomic patterns and the upregulation of L-saccharopine were also observed between the HFD-FMT and ND-FMT groups (Figure 6e-g). Subsequently, we analyzed the relationship between the relative abundance of differentially abundant bacteria (those with a relative abundance greater than 1%) and fecal L-saccharopine content using Spearman’s correlation. The findings revealed a significant positive correlation between the relative abundance of nearly all gut microbes that increased in HFD mice and the L-saccharopine content in the feces, with a pronounced effect observed in bacteria belonging to the order *Clostridiales* (Figure 6h). As evidenced by the results of our experiments, both the HFD and the HFD-FMT led to heightened intestinal permeability in the mouse subjects. Thus, we speculated that L-saccharopine accumulated in the gut would cross the compromised barrier into the intercellular space of colon epithelial cells and subsequently translocate into the systemic circulation, ultimately reaching the ovary where it affects ovarian function. To explore this, we next performed targeted metabolomics profiling of L-saccharopine levels in the serum and ovary samples from ND, HFD, ND-FMT, and HFD-FMT groups using UHPLC-MS/MS. We found that L-saccharopine levels in both serum and ovarian tissues were significantly higher in the HFD group compared to the ND group, and similar trends were observed in the FMT groups (Figure 6i-l).

To ensure that the observed differences in L-saccharopine levels between the ND and HFD donor groups were not due to discrepancies in the diets themselves, we examined L-saccharopine levels in the mouse diets and found no significant difference between the normal and high-fat diets (Figure 6m). Furthermore, we analyzed the transmitted materials obtained from the supernatant of donor mouse feces following strict dilution and filtration procedures, which also showed no significant difference in L-saccharopine levels (Figure 6n). Since L-lysine is known to catabolize into L-saccharopine,^36^ we also investigated the levels of L-lysine and found no notable differences in the diets, transmitted materials, serum, and ovarian tissues (Fig. S7). Collectively, these findings indicated that the elevated L-saccharopine may play a crucial role in HFD-induced ovarian dysfunction.

### L-saccharopine inhibited E2 synthesis by inhibiting AMPKα/MFF-mediated mitochondrial fission

**Figure 7.**
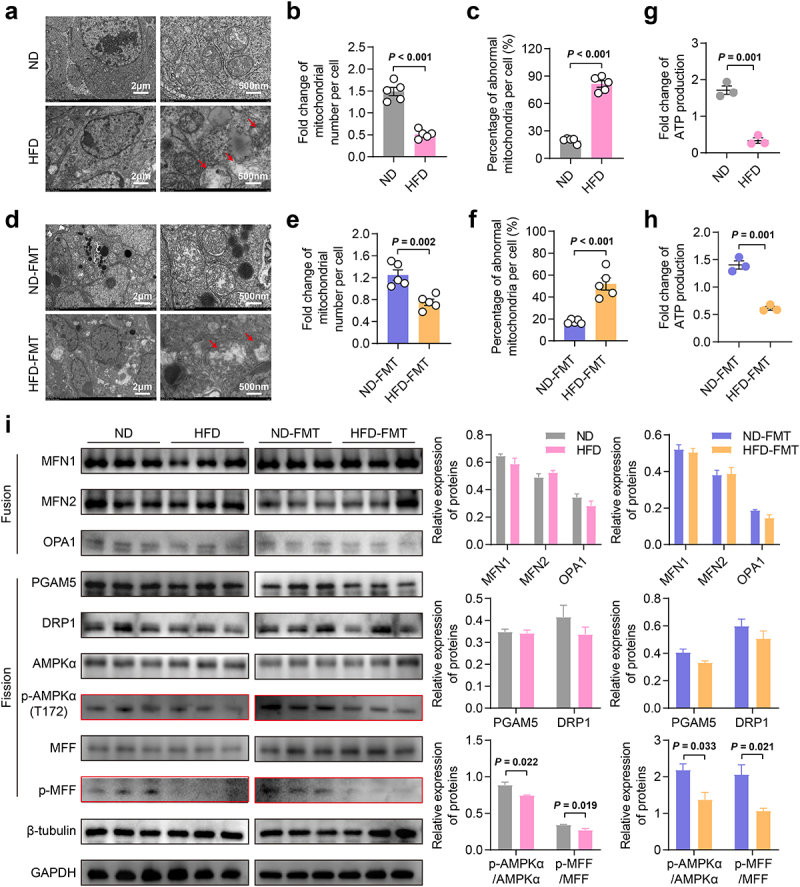
HFD and HFD-FMT induced abnormalities in ovarian mitochondrial morphology and function. a-f, Transmission electron microscopy (TEM) images showing morphological changes in mitochondria in the ovaries of mice, as well as the number and proportion of abnormal mitochondria (*n* = 5). Scale bars, 2 μm and 500 nm. Red stars indicate markedly abnormal mitochondria. g, h, Fold-change of ATP production in the ovaries of mice (*n* = 3). i, Expression of proteins critical for mitochondrial fusion and fission in the ovaries of mice by western blotting (*n* = 3). Individual values are displayed as dots, while mean ± SEM is shown as a column and error bar. Statistical significance was determined using a two-tailed unpaired Student’s *t*-test. *p* < 0.05 was considered statistically significant. ND, normal diet; HFD, high-fat diet; FMT, fecal microbiota transplantation; ATP, adenosine triphosphate.

L-saccharopine has been reported to have toxic effects on mitochondria. Its accumulation may lead to abnormal mitochondrial dynamics, ultimately disturb mitochondrial homeostasis.^37^ Furthermore, a dysregulation of mitochondrial morphology and function could affect ovarian steroidogenesis.^38,39^ Therefore, we initially examined the mitochondrial response in ovarian tissues by transmission electron microscopic (TEM) analysis. Both the HFD and HFD-FMT induced a decrease in mitochondrial count and an increase in the proportion of mitochondria with abnormal morphology, such as swelling, membrane rupture, loss of cristae, vacuolization, and giant size (Figure 7a-f). Additionally, the HFD and HFD-FMT also impaired mitochondrial function, as evidenced by reduced ATP production (Figure 7g,h). We assessed the expression of key proteins involved in mitochondrial fusion and fission using western blot analysis^40,41^ and observed that both HFD and HFD-FMT significantly reduced the phosphorylation of AMPKα and MFF (Figure 7i), which are crucial steps in the initiation of mitochondrial fission.^41^ These results suggested that L-saccharopine could disrupt mitochondrial fission by inhibiting the phosphorylation of AMPKα/MFF, perturbing the dynamic equilibrium of ovarian mitochondria and ultimately compromising ovarian function.

**Figure 8.**
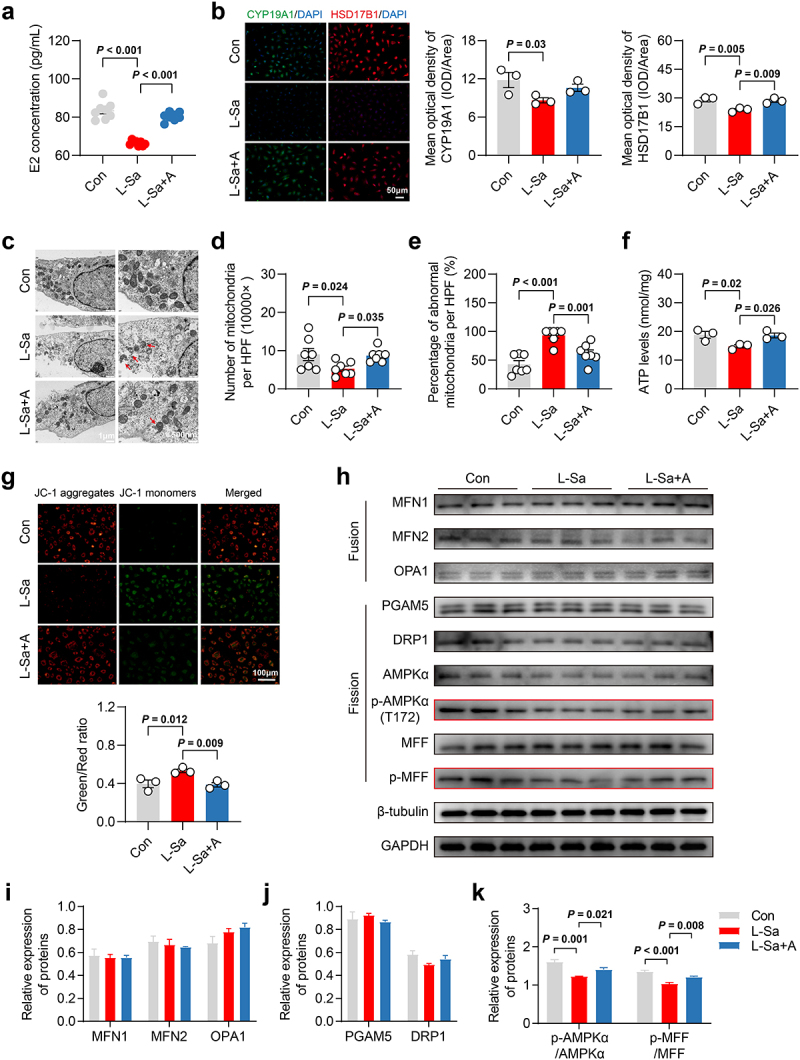
L-saccharopine inhibited E2 synthesis in mGCs *in* *vitro* by inhibiting AMPKα/MFF-mediated mitochondrial fission. a, E2 concentrations in the medium of mGCs after 48 h of intervention (*n* = 8). b, Protein expression of CYP19A1 and HSD17B1 in the mGCs after 48 h of intervention, detected by immunofluorescence (*n* = 3). Scale bar, 50 μm. c-e, Transmission electron microscopy (TEM) images of morphological changes in mGCs mitochondria after 48 h of intervention as well as the number and proportion of abnormal mitochondria (*n* = 7). Scale bars, 1 μm and 500 nm. Red stars indicate markedly abnormal mitochondria. f, Intracellular ATP levels in mGCs after 48 h of intervention (*n* = 3). g, Mitochondrial membrane potential (MMP) observed with fluorescent microscope in mGCs after after 48 h of intervention, and intensity of green and red fluorescence ratio (*n* = 3). Scale bar, 100 μm. h-k, Expression of proteins critical for mitochondrial fusion and fission in mGCs after 48 h of intervention, detected by western blotting (*n* = 3). Individual values are displayed as dots, while mean ± SEM is shown as a column and error bar. Statistical significance was determined using a one-way ANOVA, followed by LSD multiple comparisons test. *p* < 0.05 was considered statistically significant. E2, estradiol; Con, control; L-Sa, L-saccharopine; A, acadesine (AICR); HPF, high-power field; ATP, adenosine triphosphate.

To verify this effect of L-saccharopine, we performed an *in vitro* experiment using mouse granulosa cells (mGCs). We found that cell viability was significantly affected by L-saccharopine at concentrations exceeding 10 μM for 48 h and over 100 μM for 24 h (Fig. S8a-f). Targeted metabolomic assays revealed that serum L-saccharopine concentrations were below 100 nM in the ND and ND-FMT mice, while those in the HFD and HFD-FMT mice were over 100 nM. Thus, we treated cells with 100 nM L-saccharopine for 48 h (group L-Sa). There were no significant changes in cell apoptosis or proliferation (Fig. S8g-k). The concentrations of E2 were significantly lower in the culture medium of mGCs treated with L-saccharopine compared to the control group (Figure 8a), This reduction was accompanied by a notable downregulation in the expression of CYP19A1 and HSD17B1 (Figure 8b). TEM showed that L-saccharopine treatment reduced the number of mitochondria per high-power field and increased the proportion of mitochondria with abnormal morphology (Figure 8c-e). Furthermore, mitochondrial function was also significantly compromised by L-saccharopine, as evidenced by decreased ATP production (Figure 8f) and reduced mitochondrial membrane potential (MMP) (Figure 8g). We investigated its impact on critical proteins regulating mitochondrial fusion and fission, and confirmed that L-saccharopine significantly inhibited AMPKα/MFF phosphorylation (Figure 8h-k). Additionally, we tested whether the activation of AMPKα/MFF using acadesine (AICAR, 125 μM), an AMPKα activator,^42^ could ameliorate the effects of L-saccharopine on mGCs (group L-Sa+A). Encouragingly, co-treatment with AICAR and L-saccharopine significantly enhanced AMPKα/MFF phosphorylation compared to L-saccharopine alone, and markedly improved cellular E2 synthesis as well as mitochondrial morphology and function (Figure 8). Collectively, these results suggested that L-saccharopine may disrupt mitochondrial homeostasis by inhibiting AMPKα/MFF-mediated mitochondrial fission, which in turn hamper E2 synthesis in granulosa cells.

### L-saccharopine damaged mouse oocyte quality by inducing mitochondrial dysfunction

**Figure 9.**
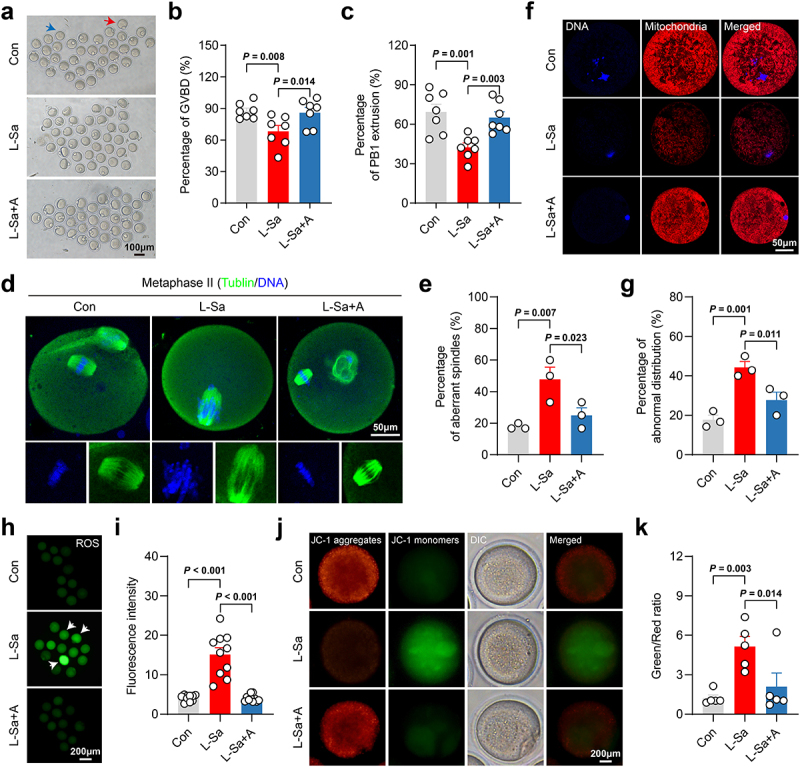
L-saccharopine inhibited oocyte maturation by inducing mitochondrial dysfunction. a, Representative images of matured oocytes cultured *in vitro* with control, L-Sa, and L-Sa + A treatments, the blue arrow indicates GVBD oocytes and the red arrow indicates PBE oocytes. Scale bar, 100 μm. b, Rate of GVBD was recorded in the three groups (*n* = 6). c, Rate of PB1 extrusion was calculated in the three groups (*n* = 6). d, Representative images of the spindle morphology and chromosome alignment at metaphase II of the three groups. Scale bar, 50 μm. e, Percentage of aberrant spindles (*n* = 3). f, Representative images of mitochondrial distribution in the three groups. Oocytes were stained with MitoTracker red to show mitochondria. Scale bar, 50 μm.g, Rate of abnormal mitochondrial distribution (*n* = 3). h, Representative images of ROS levels detected by DCFH-DA staining. Scale bar, 200 μm.I, The fluorescence intensity of ROS (*n* = 10). j, Mitochondrial membrane potential (MMP) was detected by JC-1 staining in the three groups. Scale bar, 200 μm.K, Ratio of green to red fluorescence intensity calculated in oocytes (*n* = 5). Individual values are displayed as dots, while mean ± SEM is shown as a column and error bar. Statistical significance was determined by one-way ANOVA, followed by LSD multiple comparisons test. *p* < 0.05 was considered statistically significant. Con, control; L-Sa, L-saccharopine; A, acadesine (AICAR); GVBD, germinal vesicle breakdown; PBE, polar body extrusion; PB1, first polar body; ROS, reactive oxygen species; DCFH-DA, dichlorofluorescein diacetate.

Animal experiments demonstrated that HFD and HFD-FMT resulted in deteriorated fertility in mice. Given the strong link between mitochondrial dysfunction and oocyte quality,^1^ we explored the effects of L-saccharopine on germinal vesicle (GV) oocytes, both with and without the presence of AICAR. The rates of germinal vesicle breakdown (GVBD) and polar body extrusion (PBE), which are essential developmental stages during oocyte meiosis and maturation, were significantly lower in the L-saccharopine-treated group compared to the control group (Figure 9a-c). Because meiotic arrest was mainly due to defective spindle/chromosome structure,^43^ we further tested for this in metaphase II (M II) oocytes. Immunofluorescence staining images revealed the presence of a diverse array of disorganized spindle apparatuses with misaligned chromosomes in oocytes treated with L-saccharopine (Figure 9d). Quantitative analysis demonstrated a significantly higher incidence of aberrant spindle/chromosome structures in the L-saccharopine group compared to controls (Figure 9e). These findings indicated that L-saccharopine disrupted oocyte meiosis and impeded oocyte maturation. The distribution of mitochondria changes dynamically during oocyte maturation. During the M II and subsequent stages, mitochondria are distributed throughout the cytoplasm and then allocated evenly among blastomere.^44^ However, MitoTracker staining showed an increased rate of abnormal mitochondrial distribution in the cytoplasm of M II oocytes post-treatment, predominantly characterized by their absence (Figure 9f,g), pointing to compromised mitochondrial function. Since reactive oxygen species (ROS) content and MMP are commonly used to evaluate mitochondrial function,^45^ we assessed ROS content and MMP by dichlorofluorescein diacetate (DCFH-DA) and JC-1 staining, respectively. L-saccharopine treatment led to a significant increase in ROS content (Figure 9h,i) and a decrease in MMP (Figure 9j,k), indicating mitochondrial dysfunction. Intriguingly, AICAR, which improved mitochondrial function in mGCs *in vitro*, also significantly ameliorated the impaired quality and mitochondrial dysfunction induced by L-saccharopine in mouse oocytes (Figure 9). These findings suggested that impaired oocyte quality, due to L-saccharopine-induced mitochondrial dysfunction, may become a critical mechanism by which both the HFD and HFD-induced microbiota dysbiosis impair fertility in female mice.

### L-saccharopine caused ovarian dysfunction by disrupting mitochondrial homeostasis in vivo

**Figure 10.**
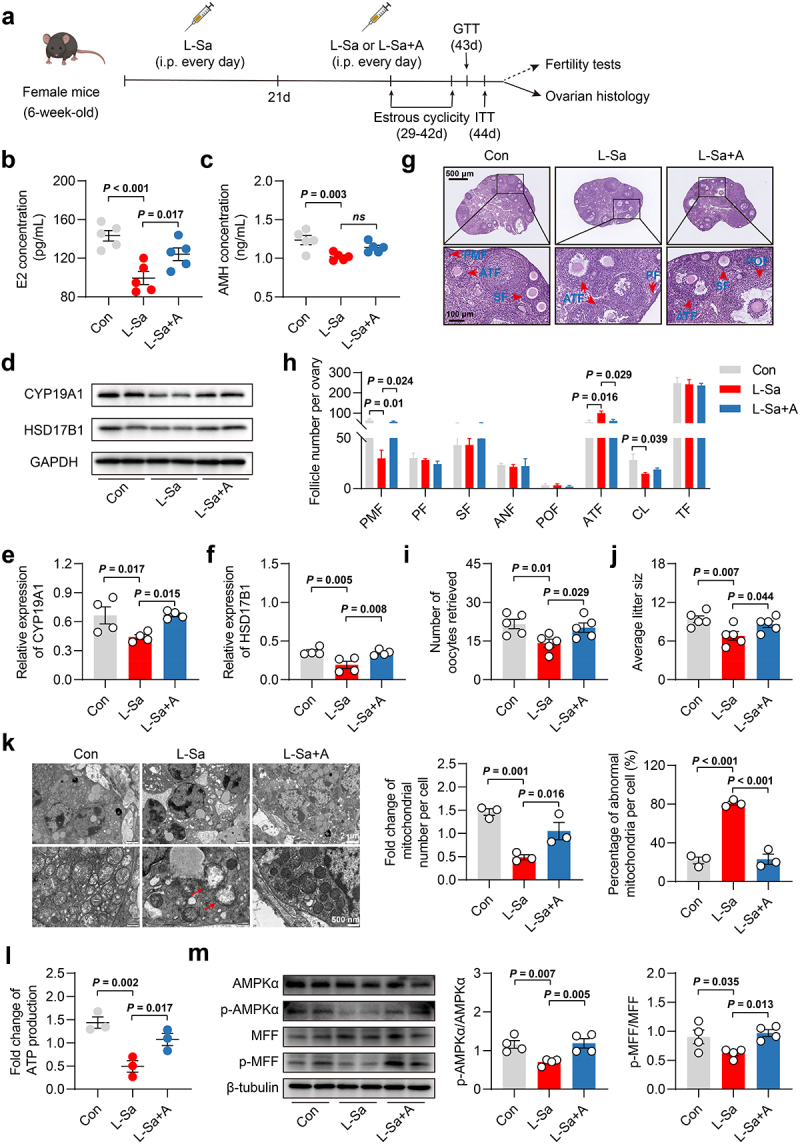
L-saccharopine induced ovarian dysfunction by disturbing mitochondrial homeostasis *in* *vivo*. a, Schematic diagram of the in *vivo* experiment. b, Serum E2 levels in mice (*n* = 5). c, Serum AMH levels in mice (*n* = 5). d-f, Protein expression of CYP19A1 and HSD17B1 in the ovary of mice by western blot (*n* = 4). g, Representative H&E staining images of mouse ovaries. Scale bars, 500 μm and 100 μm. h, Follicle counting results according to ovarian serial sections (*n* = 3). i, Number of oocytes retrieved after superovulation (*n* = 5). j, Average litter size for each female mouse (*n* = 5). k, Transmission electron microscopy (TEM) images showing morphological changes in mitochondria in the ovaries of mice, as well as the number and proportion of abnormal mitochondria (*n* = 3). Scale bars, 2 μm and 500 nm. Red stars indicate markedly abnormal mitochondria. l, Fold-change of ATP production in the ovaries of mice (*n* = 3). m, Protein expression of AMPKα, p- AMPKα, MFF, and p-MFF in the ovaries of mice by western blotting (*n* = 4). Individual values are displayed as dots, while mean ± SEM is shown as a column and error bar. Statistical significance was determined using a one-way ANOVA, followed by LSD multiple comparisons test. *p* < 0.05 was considered statistically significant. Con, control; L-Sa, L-saccharopine; A, acadesine (AICAR); GTT, glucose tolerance test; ITT, insulin tolerance test; E2, estradiol; AMH, anti-Müllerian hormone; *ns*, not significant; PMF, primordial follicle; PF, primary follicle; SF, secondary follicle; ANF, antral follicle; POF, pre-ovulatory follicle; ATF, atretic follicle; CL, corpus luteum; TF, total follicle; HPF, high-power field; ATP, adenosine triphosphate.

To further explore the potential impact of HFD-enriched L-saccharopine on ovarian function through the disruption of mitochondrial homeostasis *in vivo*, mice were administered with L-saccharopine intraperitoneally at a dosage of 2.5 mg/kg per day (Figure 10a). Over a 6-week intervention period, no significant differences were observed in body weight, glucose tolerance, or insulin resistance (Fig. S9a-e). However, the L-Sa group showed significantly decreased levels of serum E2 and AMH, along with reduced expression of CYP19A1 and HSD17B1 in the ovaries (Figure 10b-f). These mice also exhibited arrested estrous cycles and elevated serum FSH levels, though no significant differences were found in the prevalence of irregular estrous cycles and FSH levels between the L-Sa and control groups (Fig. S9f-h). Histological analysis revealed a notable decrease in the quantity of primordial follicles and corpus luteum, and a significant increase in atretic follicles in the ovaries of L-Sa mice (Figure 10g,h). Similar to the effects observed with the HFD and HFD-FMT mice, the L-Sa mice showed decreased fertility, evidenced by a notable reduction in the number of oocytes retrieved post-superovulation and the average litter size in mating test (Figure 10i,j). Moreover, abnormalities in mitochondrial morphology and function were evident in the ovaries of L-Sa mice. These included a marked decrease in mitochondrial count and an increase in the proportion of mitochondria with abnormal morphology per high power field as observed under TEM. These findings were paralleled by significant reductions in ATP production and inhibition of AMPKα/MFF phosphorylation (Figure 10k-m). Interestingly, activation of AMPKα/MFF through the administration of AICAR (10 mg/kg per day) demonstrated significant improvement in mitochondrial and ovarian dysfunction induced by L-saccharopine, as illustrated in Figure 10. These findings from the *in vivo* experiment indicated that L-saccharopine may impair ovarian function by disrupting mitochondrial homeostasis, ultimately leading to reduced fertility.

## Discussion

In the present study, we observed that the impact of HFD on ovarian function varied depending on the age at which intervention occurred, with post-puberty mice showing heightened sensitivity. This was evidenced by a reduction in ovarian reserve and endocrine dysfunction. Importantly, we confirmed that dysbiosis of the gut microbiota contributed to the deterioration of fertility and disruption of E2 synthesis caused by the HFD. Our findings also indicated that L-saccharopine disturbed mitochondrial homeostasis by inhibiting AMPKα/MFF-mediated mitochondrial fission, which subsequently impaired E2 synthesis and compromised oocyte quality. Overall, our study underscores the role of the gut microbiota-metabolite-ovary axis in mediating the negative effects of HFD on ovarian function. It suggests that targeting this axis could potentially offer therapeutic strategies to mitigate HFD-induced ovarian dysfunction. These insights pave the way for further research into interventions aimed at preserving ovarian health in the context of metabolic disturbances like HFD.

Research on ovarian function has indicated that HFD can lead to endocrine dysfunction and impaired fertility.^12–15^ However, most studies have administered the HFD shortly after weaning,^46^ without accounting for age-related effects. In our study, female mice administered with a HFD at pre-puberty, post-puberty, young adult, and middle age all showed some degree of ovarian dysfunction. Our study revealed variations in ovarian function in mice based on the age at which a HFD was initiated, with a more significant impact observed when the HFD was introduced post-puberty. The underlying cause of this variation remains uncertain. Existing literature indicates that the effects of HFD on health outcomes and the underlying mechanisms are contingent upon various factors, including age.^47,48^ For example, a study examining the effects of a HFD on cardiac structure in mice revealed age-dependent alterations, potentially attributed to varying responses of cardiomyocytes to increased fatty acid levels across different age groups.^49^ Additionally, Sakamoto *et al*. observed an age-related influence of HFD on the distribution of immunoglobulins in the lamina propria of small intestinal villi in mice.^50^ These studies provide a basis for further research into the mechanisms behind age-related disparities in HFD effects on ovarian function.

Several lines of evidence have implicated gut microbiota dysbiosis in the pathogenesis of various HFD-induced diseases, including male infertility.^10,51^ Research on polycystic ovarian syndrome has highlighted the close interplay between ovarian function and gut microbiota.^26^ However, the role of gut microbiota in HFD-induced ovarian dysfunction is still largely unknown. A recent study has found that HFD-induced endotoxemia promoted the activation of primordial follicles through macrophage infiltration and inflammatory factor secretion,^52^ suggesting that HFD may impact the ovarian reserve via a gut microbiota-mediated pathway. Our study confirmed the causal relationship between HFD-induced gut microbiota dysbiosis and ovarian dysfunction. Consistent with previous findings, we observed that HFD-induced gut microbiota dysbiosis led to a significant reduction in the number of primordial follicles,^52^ accompanied by a marked downregulation of PTEN expression and a notable increase in AKT phosphorylation in the ovarian. PTEN is an identified inhibitory factor in primordial follicle activation, its reduced expression results in decreased suppression of the downstream AKT signaling pathway, thereby promoting excessive primordial follicle activation.^29^

To further investigate the mechanisms underlying ovarian dysfunction, we performed transcriptome sequencing on the ovaries of FMT mice. This analysis revealed that disrupted ovarian steroidogenesis, particularly E2 synthesis in granulosa cells, was a major manifestation of ovarian dysfunction induced by HFD-FMT. However, the precise mechanisms by which gut microbiota communicate with the ovary remain largely unexplored. A recent study has identified a new pathway for the pathogenesis of male infertility through the gut microbiota-metabolite-testis axis.^51^ A study on AD also showed that intermittent fasting could alleviate AD through the gut microbiota-metabolite-brain axis.^53^ These studies highlight the importance of metabolites as key intermediaries between gut microbiota and extraintestinal organs.^54^ Combined with the untargeted and targeted metabolomics analyses, we confirmed that L-saccharopine was a critical metabolite in HFD-induced ovarian dysfunction. Although L-saccharopine is known to exhibit some mitochondrial toxicity,^37^ its specific effects on the ovary and female fertility have not been well characterized. Our findings showed that L-saccharopine treatment inhibited the phosphorylation of AMPKα/MFF in the ovarian tissues of HFD and HFD-FMT mice. This pathway is essential for initiating mitochondrial fission.^41^ Disruption in this pathway impedes mitochondrial fission while leaving mitochondrial fusion unaffected, leading to a reduction in mitochondrial number and quality, as observed in our TEM analysis of ovarian tissues. Mitochondria in granulosa cells are crucial for ovarian steroidogenesis and follicular development.^55^ Loss of mitochondrial function would result in suppression of E2 synthesis.^33^ Our study demonstrated that L-saccharopine blocked E2 synthesis by inhibiting AMPKα/MFF-mediated mitochondrial fission. This was confirmed through both *in vitro* and i*n vivo* experiments, as well as rescue experiments using the AMPKα activator AICAR.

Furthermore, our study revealed that L-saccharopine disrupted oocyte meiosis and maturation by inducing mitochondrial dysfunction, a condition previously linked to compromised fertility.^1^ Oocytes are highly specialized cells with substantial energy demands, relying on mitochondria to produce ATP crucial for their developmental processes.^56,57^ Any disruptions in mitochondrial function have the potential to impede meiotic progression and oocyte maturation.^57,58^ Additionally, mitochondrial dysfunction often leads to heightened oxidative stress, which is known to adversely affect oocyte quality and meiotic progression.^56,59,60^ Our observations also revealed a higher incidence of aberrant spindle/chromosome structure in oocytes treated with L-saccharopine. Previous research suggests that deviations in spindle/chromosome organization may contribute to heightened inaccuracies in chromosome segregation, culminating in aneuploidies or chromosomal irregularities within the oocyte, ultimately leading to follicular atresia .^61–63^

In addition to pathways related to ovarian steroid hormone synthesis, African trypanosomiasis, malaria, and coagulation cascade were also enriched in the KEGG analysis, suggesting a potential role of gut microbiota in modulating host immune system and inflammatory response.^64,65^ A previous study has indicated a correlation between gut microbiota and susceptibility to parasites, as well as the intensity of neuroinflammation resulting from parasitic infections.^66^ FMT may alter the immune response in mice, leading to altered expression of genes related to responses to infections such as African trypanosomiasis and malaria. Additionally, dysregulation of the gut microbiome has been linked to activation of the coagulation cascade.^67^ LPS can induce activation of the coagulation cascade through various pathways, one of which involves its interaction with Toll-like receptors.^67–69^ Our study detected activation of the TLR4 signaling pathway in the ovarian tissue of HFD-FMT mice, suggesting potential activation of the coagulation cascade in the ovary. These findings offer valuable insights for future investigations into the interplay between gut microbiota and ovarian function.

HFD represents a significant public health concern, as it is closely associated with the onset of obesity and various related health complications.^70^ Epidemiological studies have shown that obesity is linked to a reduced ovarian reserve, increased infertility, and a higher incidence of miscarriage, indicating that obesity significantly contributes to diminished fertility.^71–74^ Furthermore, animal studies have revealed that HFD can disrupt ovarian steroid hormone synthesis and follicular development through mechanisms including elevated inflammation, activation of endoplasmic reticulum stress, and compromised DNA damage repair.^16–18^ Our study, however, uncovers a novel pathway through which a HFD impairs the ovarian function, emphasizing the critical role of the gut microbiota and its metabolites. Nevertheless, further research is necessary to determine whether additional mechanisms also mediate the impact of HFD or obesity on fertility.

Our work elucidates the role of the gut microbiota-metabolites-ovary axis in mediating the detrimental effects of HFD on ovarian function. However, our study has certain inherent limitations. For instance, we were unable to identify the specific bacteria responsible for the accumulation of L-saccharopine. This limitation is primarily due to the restricted precision of 16S rRNA gene sequencing in identifying species-level details and the intricate origin of L-saccharopine in the gut. Previous research has identified up to 39 species within the phyla *Firmicutes*, *Bacteroidetes*, and *Proteobacteria* that may have the capacity to synthesize L-saccharopine,^75^ indicating a broad spectrum of potential sources for this compound. Consequently, additional studies are necessary to pinpoint the principal source of L-saccharopine accumulation. Moreover, besides its role in disrupting mitochondrial homeostasis, the possibility that L-saccharopine impairs ovarian function through other mechanisms also warrants further investigation.

In conclusion, ovarian function was most significantly compromised when a HFD was initiated post-puberty and HFD-induced gut microbiota dysbiosis was causally linked to ovarian dysfunction. The underlying mechanisms may involve an impaired intestinal mucosal barrier and metabolome remodeling, leading to L-saccharopine enrichment. L-saccharopine disrupted mitochondrial homeostasis by inhibiting AMPKα/MFF-mediated mitochondrial fission, which in turn impaired E2 synthesis and compromised oocyte quality. Our findings suggest that targeting the gut microbiota-metabolites-ovary axis may offer a promising therapeutic approach to counteract HFD-induced ovarian dysfunction.

## Methods

### Animal care and ethics

Female C57BL/6J mice (3, 5, 8, and 31 weeks old) were obtained from Beijing Huafukang Bio-Technology Co. Ltd. (Beijing, China). Mice were maintained in a specific pathogen-free environment at 22 ± 2°C, following a 12-hour light/dark cycle, with unrestricted access to food and water. After one week of free feeding and acclimation, mice with a regular estrous cycle and normal body weight (4 weeks old: 12-15 g; 6 weeks old: 15-18 g; 9 weeks old: 18-21 g; 32 weeks old: 25-28 g) were selected for the experiments. All animal experiments were reviewed and approved by the Animal Ethical Committee of Tongji Medical College, Huazhong University of Science and Technology (clearance no. 20203085).

### HFD models at different ages

To investigate whether the effect of HFD was related to the age of onset, a HFD (MD12033, 60 kcal % fat) or a ND (MD12031, 10 kcal % fat) (Table S1) was administered to female C57BL/6J mice at 4 (pre-puberty, PrP), 6 (post-puberty, PoP), 9 (young adult, You), and 32 (middle age, Mid)-weeks of age (*n* = 15 for each group). MD12033 and MD12031 were purchased from Medicience Diets Co. LTD (Yangzhou, China), the primary fat sources in these diets are lard and soybean oil. Notably, MD12033 has been widely utilized in studies pertaining to diet-induced obesity.^76–78^ After 12 weeks of intervention, mice were tested for body weight, glucose tolerance, ovarian function, and gut microbiota.

### FMT models

To explore whether a HFD can damage ovarian function by reshaping gut microbiota, female C57BL/6J mice at 6 weeks of age were randomly divided into four groups: ND (*n* = 15), HFD (*n* = 15), ND-FMT (*n* = 25), and HFD-FMT (*n* = 25). After a 4-week dietary intervention, fecal samples collected from either the ND or HFD donor groups were transplanted into the ND-FMT and HFD-FMT groups, which were maintained on a ND (see “FMT procedure”). FMT was performed twice a week for 14 weeks, during which the donor mice were consistently fed either a ND or a HFD. After 14 weeks of FMT intervention, fecal samples were collected from all groups for microbiome and metabolomics analyses. Some mice from each group were kept for mating tests (*n* = 7–12 for each group), while others were sacrificed to collect sera and tissues. For the mating tests, two female mice and one male mouse of the same age were kept in a cage, the male mouse was removed after 15 d, and pregnancy and fertility were monitored. Each female mouse was mated three times.

### FMT procedure

All mice in the two FMT groups were administered a combination of ampicillin (A8180, Solarbio, Beijing, China; 1 g/L), neomycin sulfate (N8090, Solarbio; 1 g/L), metronidazole (M8060, Solarbio; 1 g/L), and vancomycin (V8050, Solarbio; 500 mg/L) in drinking water for one week before transplantation to remove indigenous microorganisms.^79^ Fresh fecal samples collected from the ND and HFD group were diluted in sterile saline to a working concentration of 100 mg/mL, and then filtered by sterile gauze after 5 min of vortex oscillation.^10^ Transplantation into recipient mice was achieved by gavage with 200 μL of fecal-sample supernatant twice a week for 14 weeks.

### Drug treatment

L-saccharopine (L-Sa) and Acadesine (AICAR, A) were purchased from Toronto Research Chemicals (S808340; Toronto, Canada) and MCE (HY-13417; MedChem Express), respectively. Six-week-old female C57BL/6J mice were randomly divided into three groups: Control (*n* = 18), L-Sa (*n* = 18), and L-Sa+A (*n* = 18). Mice in the L-Sa and L-Sa+A groups were administered intraperitoneal injections of L-saccharopine (2.5 mg/kg dissolved in saline) daily for 6 weeks. Furthermore, the mice in the L-Sa+A group received additional intraperitoneal injections of AICAR (10 mg/kg dissolved in saline) daily for the final 3 weeks. The dosages administered were equivalent across all groups. Equivalent volumes of saline were utilized as a control in the study. Following a 6-week intervention period, a subset of mice from each experimental group were selected for mating tests (*n* = 5 per group) and superovulation experiments (*n* = 5 per group), while the remaining mice were euthanized for serum and tissue collection. To assess the number of oocytes retrieved post-superovulation, mice were administered intraperitoneal injections of 10 IU pregnant mare serum gonadotropin (PMSG; P9970; Solarbio, Beijing, China), followed by a subsequent injection of 10 IU human chorionic gonadotropin (hCG) 48 hours after PMSG priming. Oocytes were retrieved from the ampullae of the oviduct 13.5 hours after administrating hCG.

### Intestinal permeability assay in vivo

The *in vivo* assay for gut permeability was conducted according to previously established methods.^80^ Mice were orally administered 600 mg/kg of FITC-labeled dextran (average molecular weight 4000; FD 40, Sigma) after a 5-hour fasting period. Serum samples were collected at specified time intervals and analyzed using a microplate reader (SpectraMax Paradigm, Molecular Devices, Sunnyvale, CA, USA) at wavelengths of 485 nm (excitation) and 535 nm (emission).

### Estrous cycle monitoring

The estrous cycle was monitored for 14 d consecutively before mice were sacrificed. Vaginal smears were taken at 9 am every day and used to determine the stage of the estrous cycle by microscopic identification of the predominant cell type following hematoxylin and eosin (H&E) staining. In mice, the estrous cycle is characterized by four distinct stages, namely proestrus, estrus, metestrus, and diestrus, which occur sequentially and recur every 4 to 5 days.^81^ Mice were classified as exhibiting regular cycling patterns if they consistently demonstrated cycles lasting 4–5 days. Those with cycle lengths falling outside of this range, as well as mice lacking discernible cycles, were classified as irregularly cycling.^82^ The proestrus phase consists mainly of round nucleated cells. The estrus phase contains abundant anucleated cornified epithelial cells. At metestrus, numerous leukocytes and a small number of large nongranular and anucleated cornified epithelial cells are observed. Diestrus contains prominent polymorphonuclear leukocytes and a few epithelial and cornified cells.^83^

### Glucose tolerance test (GTT) and insulin tolerance test (ITT)

Mice were subjected to a GTT and ITT before sacrifice. During the GTT, each mouse received 2 g/kg body weight of glucose via intraperitoneal injection after 12 hours of fasting.^10^ During the ITT, each mouse received a single intraperitoneal injection of insulin (0.75 IU/kg body weight) after 4 hours of fasting.^84^ Blood samples were collected before injection (0 min) and at 15, 30, 60, 90, and 120 min after injection. Glucose levels were determined using a glucometer (Accu-Chek Performa, Roche Diagnostics, Mannheim, Germany).

### Determination of serum sex hormones, LPS, and cytokines

All mice underwent estrous cycle testing at 9 am, and blood collection was conducted at approximately 10 am following confirmation of diestrus phase. The serum levels of FSH, E2, P4, AMH, LPS, TNF-α, IL-1β, and IL-6 were detected by enzyme-linked immunosorbent assay (ELISA) according to the manufacturer’s instructions (CSB-E06871m, CSB-E05109m, CSB-E05104m, CSB-E13156m, CSB-E13066m, CSB-E04741m, CSB-E08054m, and CSB-E04639m, respectively; Cusabio Technology LLC, Houston, TX, USA). The detection range, sensitivity, intra-assay, and inter-assay precision of the ELISA kits utilized in our research were presented in Table S2.

### Measurement of serum lipids

Serum total cholesterol (TC), triglyceride (TG), low-density lipoprotein cholesterol (LDL-C), and high-density lipoprotein cholesterol (HDL-C) levels were measured using an Automatic Biochemical analyzer (BK-280, BIOBASE, Jinan, China).

### Follicle counting

Follicle counting was performed as previously described.^85^ Briefly, three to five ovaries from distinct individuals in each group were selected for follicle counting. After sacrifice, the ovaries of each mouse were quickly collected, fixed in 4% paraformaldehyde, dehydrated in 70% ethanol, and embedded in paraffin. The ovaries were longitudinally sectioned into 4 μm sections. Four sections were mounted on a glass slide. Every fifth slide was stained with H&E and analyzed under a light microscope (CX41; Olympus, Tokyo, Japan). Only follicles containing oocytes were counted. Representative images of typical follicles were recorded using the Pannoramic SCAN (3DHISTECH, Budapest, Hungary).

### Sirius red staining

Four representative ovarian sections from each group were chosen to assess fibrosis level by Sirius Red staining. The ovaries were deparaffinized with xylene I for 20 min, xylene II for 20 min, 100% ethanol I for 5 min, 100% ethanol II for 5 min, and 75% ethanol for 5 min before being rinsed with tap water. The slices were stained with Sirius Red solution (G1018, Sirius Red staining kit, Servicebio) for 8 min, dehydrated with two changes of anhydrous ethanol, and placed in clean xylene for 5 min. The stained slices were sealed with neutral gum, and images were recorded using the Pannoramic SCAN (3DHISTECH) and analyzed using SlideViewer (3DHISTECH). All assessments were performed using the Image-Pro Plus software (IPP 6.0; Rockville, MD, USA).

### Masson trichrome staining

Four representative ovarian sections from each group were selected and reevaluated for the degree of fibrosis using Masson trichrome staining. Following deparaffinization, as previously described for Sirius Red staining, the slices were soaked overnight in Masson A (G1006; Masson staining kit; Servicebio) and subsequently rinsed with tap water. Masson B and Masson C were mixed in a 1:1 ratio to prepare the Masson solution. The slices were then stained with this solution for 1 min and rinsed with tap water. Differentiation was achieved with 1% hydrochloric acid alcohol, followed by another rinse with tap water. The slices were then soaked in Masson D for 6 min, rinse with tap water; soaked in Masson E for 1 min, and Masson F for 20–30 s, followed by a rinse in 1% glacial acetic acid. The slices underwent dehydration through two changes of anhydrous ethanol, were placed in clean xylene for 5 min, and sealed with neutral gum. Observation and analysis were conducted similarly to the Sirius Red staining process.

### Periodic acid-Schiff-Alcian Blue (PAS-AB) staining

Mice colons were collected, fixed in 4% paraformaldehyde, dehydrated in 70% ethanol, and embedded in paraffin. The colons were transversely sectioned into 5 μm slices. After deparaffinization, as described for Sirius Red staining, the slices were stained with AB-PAS C (G1049; PAS-AB staining kit; Servicebio) for 15 min, and rinsed with tap water until the runoff was colorless. Subsequently, the slices were stained with AB-PAS B for 15 min, followed by rinsing with tap water and then twice with distilled water. AB-PAS A was brought to room temperature and used to stain the slices for 30 min in the dark, followed by a 5-minute rinse. The slices were dehydrated in three changes of 100% ethanol, each for 5 min, cleared in two changes of xylene, each also for 5 min, and finally sealed with neutral gum. Images of the stained slices were captured using the Pannoramic SCAN (3DHISTECH), and the thickness of the colonic mucosa was measured using SlideViewer (3DHISTECH).

### Immunohistochemistry (IHC)

Mice ovarian or colonic sections were processed for immunohistochemical analysis using routine IHC procedures^85^ and the following primary antibodies: rabbit anti-AMH (14461–1-AP; Proteintech, Wuhan, China; 1:100), rabbit anti-CYP17A1 (14447–1-AP; Proteintech; 1:1,600), rabbit anti-CYP19A1 (A2161; ABclonal, Wuhan, China; 1:800), rabbit anti-HSD17B1 (25334–1-AP; Proteintech; 1:800), and mouse-anti MYD88 (67969–1-lg; Proteintech; 1:100). Horseradish peroxidase (HRP)-conjugated secondary antibodies included goat anti-rabbit IgG (H+L) (ab205718; Abcam, Cambridge, UK; 1:2,000), and goat anti-mouse IgG (H+L) (ab205719; Abcam; 1:2,000). Section scanning and observation were performed in the same manner as described above. IHC scores based on the integrated optical density (IOD)/area were calculated using Image-Pro Plus software (IPP 6.0) and used to evaluate the relative expression of related proteins.

### Cell culture and reagents

Ovaries from 21-d-old C57BL/6J female mice were harvested for mouse granulosa cells (mGCs), as previously described.^86^ The human ovarian granulosa cell lines KGN and COV434 were obtained from Procell Life Science & Technology Co. Ltd. (Wuhan, China). Primary mGCs, KGN, and COV434 cells were cultured in McCoy’s 5a (M4892; Sigma-Aldrich, St. Louis, MO, USA), DMEM/F12 (PYG0004; Bosterbio, Wuhan, China), and DMEM (12800, Gibco, CA, USA), respectively. All media contained 5% fetal bovine serum, 100 IU/mL penicillin, 100 μg/mL streptomycin sulfate, and 10^−7^ M androstenedione. The cells were maintained in an atmosphere of 37°C and 5% CO2/95% humidified air. In the *in vitro* experiments, L-Sa, and AICAR were dissolved in the medium of the corresponding treatment group.

### Cell viability assay

Cells were seeded in a 96-well cell culture plate at a density of 5 × 10^3^ and treated with L-Sa (1 nM, 10 nM, 100 nM, 1 μM, 10 μM, 100 μM, and 1 mM). To evaluate the effects of L-Sa on cell proliferation, cell viability was assessed using a CCK8 kit (A311–01; Vazyme, Nanjing, China) after 24 h and 48 h of culture, according to the manufacturer’s instructions.

### EdU assay

The effects of L-Sa (100 nM for 48 h) on cell proliferation were confirmed using an EdU Cell Proliferation Image Kit (KTA2030; Abbkine Scientific, Wuhan, China) according to the manufacturer’s instructions. The proportion of EdU-positive cells was calculated based on our previous criteria.^87^

### Flow cytometry

The effects of L-Sa (100 nM for 48 h) on apoptotic activity were detected using an annexin-Ⅴ-FITC apoptosis detection kit (556547; BD Pharmingen, San Diego, CA, USA) according to the manufacturer’s instructions. The percentage of apoptotic cells (from 10,000 cells) was measured using a flow cytometer (BD Pharmingen).

### Measurement of E2 levels in the cell medium

After L-Sa (100 nM for 48 h), or L-Sa (100 nM for 48 h) +A (125 μM for 48 h) treatment, E2 levels were measured in the cell supernatant by ELISA (CEA461Ge; Cloud-Clone Corp, TX, USA) according to the manufacturer’s instructions.

### Immunofluorescence (IF)

Following deparaffinization and hydration, mouse ovarian sections were incubated with primary antibodies overnight at 4°C. The cells were fixed with 4% paraformaldehyde for 20 min at room temperature and washed with phosphate-buffered saline (PBS). After permeabilizing the membrane and blocking, the cells were incubated with the following primary antibodies at 4°C overnight: rabbit anti-STAR (12225–1-AP; Proteintech; 1:100) and mouse anti-TLR4 (ab22048; Abcam; 1:100), rabbit anti-CYP19A1 (ab18995; Abcam; 1:200), and rabbit anti-HSD17B1 (A10839; ABclonal; 1:200). On the following day, the immunofluorescent signals were labeled with the following fluorescent secondary antibodies: FITC goat anti-rabbit IgG (H+L) (AS011; ABclonal; 1:50), FITC donkey anti-rabbit IgG (H+L) (AS042; ABclonal; 1:50), FITC goat anti-mouse IgG (H+L) (AS001; ABclonal; 1:50), and Cy3 goat anti-rabbit IgG (H+L) (AS007; ABclonal; 1:100). Images were recorded using the Pannoramic SCAN (3DHISTECH) and analyzed using SlideViewer (3DHISTECH). Fluorescence intensity was calculated using Image-Pro Plus software (IPP 6.0) and used to evaluate the relative expression of related proteins.

### Quantitative real-time polymerase chain reaction (qRT-PCR)

Total RNA was extracted from mouse ovarian tissues using TRIzol reagent (10296101; Invitrogen, Camarillo, CA, USA). We reverse-transcribed 1 µg of RNA into cDNA using a reverse transcription kit (R223–01; Vazyme). Quantitative PCR was performed using ChamQ Universal SYBR qPCR Master Mix (Q711–02; Vazyme) in a CFX96 real-time PCR system (Bio-Rad, Hercules, CA, USA). For each sample, 2^−ΔΔCt^ was calculated to represent gene expression. The primer sequences are listed online in Table S3.

### Western blot analysis

Total protein was extracted from snap-frozen ovarian and colonic tissues. A bicinchoninic acid (BCA) protein assay kit (P0012; Beyotime, Shanghai, China) was used to determine protein concentrations. The proteins were separated by 10% sodium dodecyl sulfate polyacrylamide gel electrophoresis, and transferred to a polyvinylidene fluoride membrane. The membranes were blocked with 5% bovine serum albumin (BSA) for 1 h at room temperature and incubated with the following primary antibodies overnight at 4°C: rabbit anti-Occludin (A2601; ABclonal; 1:1,000), rabbit anti-Claudin 1 (A2196; ABclonal; 1:1,000), rabbit anti-ZO-1 (A0659; ABclonal; 1:1,000), rabbit anti-MUC 2 (A4767; ABclonal; 1:1,000), rabbit anti-PTEN (A19104; ABclonal; 1:1,000), rabbit anti-p-AKT (4060S; Cell Signaling Technology, Danvers, MA, USA; 1:1,000), rabbit anti-AKT (A18675; ABclonal; 1:1,000), rabbit anti-p-RPS6 (AP1328; ABclonal; 1:10,000), rabbit anti-RPS6 (A11874; ABclonal; 1:1,000), rabbit anti-COL1A1 (A22090; ABclonal; 1:500), rabbit anti-TGF-β (A16640; ABclonal; 1:500), rabbit anti-STAR (12225–1-AP; Proteintech; 1:500), rabbit anti-CYP17A1 (14447–1-AP; Proteintech; 1:500), rabbit anti-CYP19A1 (A12238; ABclonal; 1:500), rabbit anti-HSD17B1 (25334–1-AP; Proteintech; 1:1,000), rabbit anti-NF-κB p65/RelA (A19653; ABclonal; 1:2,000), rabbit anti-Phospho-NF-κB p65/RelA-S536 (AP0124; ABclonal; 1:2,000), rabbit anti-MFN1 (13798–1-Ap; Proteintech; 1:500), rabbit anti-MFN2 (12186–1-Ap; Proteintech; 1:2,000), rabbit anti-OPA1 (27733–1-Ap; Proteintech; 1:1,000), rabbit anti-DRP1 (12957–1-Ap; Proteintech; 1:1,000), rabbit anti-PGAM5 (28445–1-Ap; Proteintech; 1:2,000), rabbit anti-AMPKα (10929–1-Ap; Proteintech; 1:1,000), rabbit anti-p-AMPKα (#2535; Cell signaling technology; 1:1,000), rabbit anti-MFF (17090–1-Ap; Proteintech; 1:4,000), rabbit anti-p-MFF (#AF2365; Affinity; 1:500), rabbit anti-Beta Tubulin (10094–1-AP; Proteintech; 1:2,000), and rabbit anti-GAPDH (A19056; ABclonal; 1:10,000). The next day, the membranes were rewarmed for 1 h and washed three times in TBST. HRP-conjugated anti-rabbit IgG (G1213; Servicebio; 1:3,000) was incubated with the membranes as a secondary antibody for 1 h at 37°C. Immunoblots were visualized with enhanced chemiluminescence reagents (Bio-Rad). Protein bands were captured using a ChemiDoc XRS+ Imaging system (Bio-Rad) and quantified by densitometry image analysis using ImageJ Software (version 6.0.1, National Institutes of Health, Bethesda, MD, USA).

### TEM

Mouse ovaries were cut into ~1 mm^3^ sections and fixed. After dehydration in an ascending ethanol series, the tissues were permeabilized, embedded and sectioned (60–80 μm). The cells were scraped off the plates and centrifuged at 800 *rpm* for 5 min, and the supernatant was discarded. The cells were resuspended in fixing solution at 4°C for 4 h. The cells were transferred to centrifuge tubes and spun to obtain cell pellets. The cell pellet was embedded in 1% agarose and washed three times in 0.1 M PBS for 15 min each. Next, the cells were post-fixed with 1% OsO4 in 0.1 M PBS (pH 7.4) for 2 h at room temperature. OsO4 was removed, and the cells were rinsed three times in 0.1 M PBS (pH 7.4) for 15 min each. The cells were then dehydrated with 50%, 70%, 80%, 90%, and 95% ethanol for 15 min each. Next, the cells were subjected to two changes in 100% ethanol for 15 min each, and two changes in acetone for 15 min each. After infiltration and embedding, the samples were cut into ultrathin sections (60–80 nm) using an ultramicrotome (Leica UC7; Leica, Nussloch, Germany). The sections were dried overnight at room temperature after uranium-lead double staining and images were obtained by TEM (HT7700, HITACHI, Tokyo, Japan).

### MMP detection

Cellular MMP was detected using an MMP assay kit (JC-1) (KTA4001, Abbkine Scientific) according to the manufacturer’s instructions. Images were acquired using CellSens Dimension software (Olympus, Tokyo, Japan). The fluorescence intensity of mitochondrial JC-1 monomers and aggregates was calculated using Image-Pro Plus software (IPP 6.0), and the mean fluorescence intensity ratio of green to red light was calculated.

### ATP level measurement

ATP levels in cells and ovarian tissues were measured using an ATP assay kit (S0026, Beyotime) according to the manufacturer’s instructions. Luminescence was measured using a Gen5 Microplate reader (BioTek, Winooski, VT, USA). To avoid errors caused by different protein amounts, protein concentration was determined using the BCA assay kit (P0012, Beyotime).

### Oocyte collection and in vitro maturation

Germinal vesicle (GV) oocytes were collected by manually rupturing antral follicles from 21-d-old female BALB/C mice. Cumulus cells were removed by repeated mouth pipetting. For *in vitro* maturation, GV oocytes were cultured in M2 medium at 37°C in an atmosphere of 5% CO2 for 16–18 h. Oocytes were observed under an inverted microscope to determine their maturation according to the discharge of the first polar body.

### Immunofluorescence and confocal microscopy

Oocytes were fixed in 2% paraformaldehyde in PBS (4% paraformaldehyde + 0.5% BSA) for 30 min and washed twice for 10 min with PBS containing 0.5% BSA. The oocytes were incubated in PBS containing 0.5% BSA and 0.2% Triton X-100 for 45–60 min and incubated with anti-α-tubulin antibody (1:200 dilution, ABclonal, AC012) at 4°C overnight. After rewarming, they were washed twice with PBS containing 0.5% BSA for 10 min. Next, the oocytes were incubated with FITC-conjugated goat anti-rabbit IgG (Servicebio, GB22031) for 1 h at 37°C, counterstained with DAPI for 10 min, and mounted on glass slides for observation by a laser scanning confocal microscope.

For mitochondrion staining, oocytes were cultured in M2 medium containing 200 nM Mito-Tracker Red CMXRos (C1035, Beyotime) for 30 min at 37°C. After washing three times with fresh M2 medium for 20 min each, oocytes were mounted on glass slides for observation by a laser scanning confocal microscope.

### ROS detection

ROS production was evaluated using a ROS Assay Kit (Beyotime, S0033S). For dichlorofluorescein (DCFH) staining, oocytes were incubated with the oxidation-sensitive fluorescent probe (10 mm) for 30 min at 37°C in PBS containing 0.5% BSA. Oocytes were finally washed three times in PBS containing 0.5% BSA and placed on glass slides for observation by fluorescence microscopy.

### 16S rRNA gene sequencing

Genomic DNA was extracted from mouse fecal samples using an OMEGA Stool DNA Kit (D4015, Omega, Norcross, GA, USA) according to the manufacturer’s instructions. V3-V4 hypervariable regions of 16S rRNA gene were amplified using PCR (98°C for 30 s, followed by 35 cycles at 98°C for 10 s, 54°C for 30 s, and 72°C for 45 s, and a final extension at 72°C for 10 min) with slightly modified versions of the primers 341F (5’-CCTACGGGNGGCWGCAG-3’) and 805 R (5’-GACTACHVGGGTATCTAATCC-3’). Amplicon quality was visualized using 1.5% agarose gel electrophoresis. Target fragments were recovered using an AxyPrep PCR Cleanup Kit. The PCR product was further purified using a Quant-iT PicoGreen dsDNA Assay Kit. The library was prepared using a Promega QuantiFluor fluorescence quantification system. The pooled library was loaded onto an Illumina platform using a paired-end sequencing protocol (2 × 250 bp).

Paired-end reads were assigned to the samples based on their unique barcode and the barcode and primer sequence were truncated. The paired-end reads were merged using FLASH (v1.2.8). Quality filtering of the raw reads was performed under specific filtering conditions to obtain high-quality clean tags according to fqtrim (v0.94). Chimeric sequences were filtered using Vsearch software (v2.3.4). After dereplication using DADA2, we obtained a feature table and sequence. Alpha and beta diversity indices were calculated using QIIME2, in which the same number of sequences was extracted randomly by reducing the number of sequences to the minimum of some samples; relative abundance was determined according to bacterial taxonomic classification. The sequence alignment of species annotation was performed using BLAST and the alignment databases SILVA and NT-16S. LEfSe was performed to detect significantly different abundances for designated taxa of the gut microbiome using a Kruskal – Wallis rank sum test, and bioinformatics analysis was performed using OmicStudio (http://www.omicstudio.cn/tool).

### RNA sequencing

Total RNA was isolated and purified using TRIzol reagent (Invitrogen, Carlsbad, CA, USA) following the manufacturer’s instruction. The amount and purity of RNA in each sample were quantified using NanoDrop ND-1000 (NanoDrop, Wilmington, DE, USA). RNA integrity was assessed using Bioanalyzer 2100 (Agilent Technologies, Santa Clara, CA, USA) with RIN number > 7.0, and confirmed using electrophoresis on a denaturing agarose gel. Poly (A) RNA was purified from 1 μg of total RNA using Dynabeads Oligo (dT)25 -61,005 (Thermo Fisher Scientific, Carlsbad, CA, USA) with two rounds of purification. The poly(A) RNA was fragmented into small pieces using a Magnesium RNA Fragmentation Module (e6150; New England Biolabs (NEB), Ipswich, MA, USA) at 94°C for 5–7 min. The cleaved RNA fragments were reverse-transcribed to cDNA using SuperScript II Reverse Transcriptase (1896649; Invitrogen, USA), and the cDNA was used to synthesize U-labeled second-stranded DNAs with *E. coli* DNA polymerase I (m0209; NEB), RNase H (m0297; NEB), and dUTP Solution (R0133; Thermo Fisher Scientific). An A-base was added to the blunt ends of each strand to prepare them for ligation to the indexed adapters. Each adapter contained a T-base overhang to ligate the adapter to the A-tailed fragmented DNA. Single- or dual-index adapters were ligated to the fragments and size selection was performed using AMPureXP beads. After heat-labile UDG enzyme (m0280; NEB) treatment of the U-labeled second-stranded DNAs, the ligated products were amplified using PCR under the following conditions: initial denaturation at 95°C for 3 min; eight cycles of denaturation at 98°C for 15 s, annealing at 60°C for 15 s, and extension at 72°C for 30 s; and a final extension at 72°C for 5 min. The average insert size of the final cDNA library was 300 ± 50 bp. Finally, we performed 2 × 150bp paired-end sequencing (PE150) on an Illumina Novaseq 6000 (LC-Bio Technology, Hangzhou, China), following the manufacturer’s recommended protocol.

We used fastp software (https://github.com/OpenGene/fastp) to verify sequence quality and remove reads containing adaptor contamination, low-quality bases, and undetermined bases with the default parameter. HISAT2 (https://ccb.jhu.edu/software/hisat2) was used to map the reads to the reference genome of *Mus musculus*. The mapped reads of each sample were assembled using StringTie (https://ccb.jhu.edu/software/stringtie) with the default parameters. Next, all transcriptomes from all samples were merged to reconstruct a comprehensive transcriptome using gffcompare (https://github.com/gpertea/gffcompare/). After generating the final transcriptome, StringTie was used to estimate the expression of all the transcripts fragments per kilobase of transcript per million mapped reads. The differentially expressed mRNAs were selected with a fold change > 2 or < 0.5 and with a parametric F-test comparing nested linear models (*p* < 0.05) using the R package edgeR (https://bioconductor.org/packages/release/bioc/html/edgeR.html). Bioinformatic analysis was performed using OmicStudio (http://www.omicstudio.cn/tool).

### Metabolomics profiling

Metabolite extraction was performed using a 50% methanol buffer after frozen fecal samples were thawed on ice. Briefly, 20 μL of the sample was extracted with 120 μL of precooled 50% methanol, vortexed for 1 min, and incubated at room temperature for 10 min before being stored overnight at ‐20°C. After centrifugation at 4,000×*g* for 20 min, supernatants were transferred to new 96‐well plates and stored at ‐80°C prior to liquid chromatography-mass spectrometry (LC-MS) analysis. Pooled quality control (QC) samples were prepared by combining 10 μL of each extraction mixture.

All samples were acquired using the LC‐MS system. An UHPLC system (SCIEX, UK) was used for chromatographic separations. An ACQUITY UPLC T3 column (100 mm × 2.1 mm, 1.8 µm, Waters, UK) was used for reversed-phase separation. The column oven was maintained at 35°C. The mobile phase consisted of solvent A (0.1% formic acid in water) and solvent B (0.1% formic acid in acetonitrile) at a flow rate of 0.4 mL/min. The gradient elution conditions were 0 ~ 0.5 min, 5% B; 0.5 ~ 7 min, 5% to 100% B; 7 ~ 8 min, 100% B; 8 ~ 8.1 min, 100% to 5% B; 8.1 ~ 10 min, 5%B. Each sample injection volume was 4 µL.

The metabolites eluted from the column were detected using a high‐resolution tandem mass spectrometer TripleTOF5600plus (A-TOF; SCIEX, Macclesfield, UK). The Q‐TOF was operated in both positive and negative ion modes. Curtain gas was set at 30 PSI, ion source gases were set at 60 PSI, and the interface heater temperature was maintained at 650°C. For the positive and negative ion modes, the ion spray voltage floating was set at 5000 V and ‐4500 V, respectively. Data were acquired in the IDA mode. The TOF mass range was 60–1200 Da. Survey scans were acquired in 150 ms and up to 12 product ion scans were collected if the threshold exceeded 100 counts per second and at a 1+ charge‐state. The total cycle time was fixed at 0.56 s. Four-time bins were summed for each scan at a pulse frequency value of 11 kHz by monitoring the 40 GHz multichannel TDC detector with four‐anode/channel detection. Dynamic exclusion was set for 4 s. During acquisition, the mass accuracy was calibrated after every 20 samples. To evaluate the stability of the LC‐MS system during acquisition, a QC sample was acquired after every 10 samples.

XCMS software was used to acquire MS data pretreatments, including peak picking, peak grouping, retention time (RT) correction, second peak grouping, and isotope and adduct annotations. LC-MS raw data files were converted to the mzXML format and processed using R software with XCMS, CAMERA, and MetaX. Each ion was identified by combining RT and exact molecular mass (m/z) data. The intensity of each peak was recorded, and a three-dimensional matrix containing arbitrarily assigned peak indices (RT‐m/z pairs), sample names (observations), and ion intensity information (variables) was generated.

The metabolites were annotated online using the KEGG and HMDB databases by matching the m/z of the samples with that of the database. If the mass difference between the observed and database values was less than 10 ppm, the metabolite was annotated and the molecular formula was identified and validated using isotopic distribution measurements. We also validated the identified metabolites using an in‐house fragment spectrum library of metabolites.

The peak data were further preprocessed using MetaX. Features found in less than 50% of the QC samples or 80% of the biological samples were removed, and the remaining peaks with missing values were imputed with the k‐nearest neighbor algorithm to further improve data quality. Principal component analysis (PCA) was performed for outlier detection and batch effect evaluation using a pre‐processed dataset. QC‐based robust LOESS signal correction was fitted to the QC data based on the order of injection to minimize intensity drift over time. The relative standard deviations of the metabolic features were calculated for all QC samples, and those > 30% were removed. Student’s *t*‐tests were conducted to detect differences in metabolite concentrations between two phenotypes. The *P*-value was adjusted for multiple tests using an FDR (Benjamini – Hochberg). Supervised partial least squares discriminant analysis (PLS‐DA) was conducted using MetaX to discriminate the different variables between groups. VIP values were calculated, and a cut‐off value of 1.0 was used to select important features. Bioinformatic analysis was performed using OmicStudio (http://www.omicstudio.cn/tool).

### Targeted L- saccharopine quantification

For ovarian tissue samples, an aliquot of each sample was precisely weighed and transferred to a 2 mL Eppendorf tube. Next, 200 μL of a precooled (−20 °C) methanol-water solution (3:1 ratio) was added, and the samples were vortexed for 30 s. They were then homogenized at 38 hz for 4 min and sonicated for 5 min in an ice-water bath. This homogenization and sonication cycle was repeated three times. Afterward, the samples were incubated at −20°C for 1 h and centrifuged at 12,000 *rpm* and 4°C for 15 min. A 50 μL of the supernatant was diluted for 10 times for LC-MS/MS analysis.

For serum samples, a 20 μL aliquot of each sample was transferred to a 1.5 mL Eppendorf tube. After adding 60 μL of methanol, the samples were vortexed for 30 s, incubated at −20°C for 1 h, and then centrifuged at 12,000 *rpm* and 4°C for 15 min. A 50 μL of the supernatant was diluted for 10 times for LC-MS/MS analysis.

For feed samples, an aliquot of each sample was precisely weighed and transferred to a 2 mL Eppendorf tube. After adding 1,000 μL of a precooled (−20 °C) methanol-water solution (3:1 ratio), the samples were vortexed for 30 s, homogenized at 38 hz for 4 min, and sonicated for 5 min in an ice-water bath. This homogenization and sonication cycle was repeated three times, followed by incubation at −20°C for 1 h and centrifugation at 12,000 *rpm* and 4°C for 15 min. A 40 μL aliquot of the clear supernatant was then transferred to an auto-sampler vial for LC-MS/MS analysis.

For feces samples, a 20 μL aliquot of each sample was transferred to an Eppendorf tube. Following the addition of 60 μL of methanol, the samples were vortexed for 30 s. They were then incubated at −20°C for 1 h and centrifuged at 12,000 *rpm* and 4°C for 15 min. A 50 μL of the supernatant was diluted for 10 times for LC-MS/MS analysis.

Stock solutions were individually prepared by dissolving or diluting each standard substance to a final concentration of 1 mmol/L. A 100 μL aliquot from each stock solution was transferred to a 10 mL volumetric flask to create a mixed working standard solution. This solution was then used to prepare a series of calibration standards through stepwise dilution.

The UHPLC separation was performed using an Agilent 1290 Infinity II series UHPLC System (Agilent Technologies), equipped with an Agilent ZORBAX Eclipse Plus C18 (2.1 mm × 150 mm, 1.8 μm, Agilent Technologies, USA). Mobile phase A consisted of 1% formic acid in water, while mobile phase B was acetonitrile. The flow rate was set at 300 μL/min, the column temperature at 35°C, the auto-sampler temperature at 10°C, and the injection volume was 1 μL.

An Agilent 6495 triple quadrupole mass spectrometer (Agilent Technologies), equipped with an AJS electrospray ionization (AJS-ESI) interface, was used for assay development. The typical ion source parameters included a capillary voltage of +3000 V, a nozzle voltage of + 1500/V, a gas (N2) temperature of 250°C, a gas (N2) flow of 11 L/min, a sheath gas (N2) temperature of 400°C, a sheath gas flow of 12 L/min, and a nebulizer pressure of 35 psi.

The MRM parameters for each targeted analyte were optimized by directly injecting the standard solutions into the API source of the mass spectrometer. At least two MRM transitions (i.e., the Q1/Q3 pairs) per analyte were obtained, and the two most sensitive transitions selected for use in MRM scan mode to optimize the collision energy for each pair. Among the transitions, the Q1/Q3 pairs exhibiting the highest sensitivity and selectivity were for quantitative monitoring. Additional transitions served as qualifiers to confirm the identity of the target analytes.

Calibration solutions were analyzed using UPLC-MRM-MS/MS with the methods described above. Linear regression via the least squares method was employed to construct the standard curve. The calibration solutions were stepwise diluted, each with a dilution factor of 2, and then subjected to UHPLC-MRM-MS analysis. Signal-to-noise ratios (S/N) were utilized to determine the limits of detection (LODs) and lower limits of quantitation (LLOQs). The LODs and LLOQs were defined as the analyte concentrations resulting in peaks with S/N ratios of 3 and 10, respectively, according to the US FDA guidelines for bioanalytical method validation.

Precision of the quantitation was measured by the relative standard deviation (RSD) from analytical replicates of a QC sample. Accuracy was measured by the analytical recovery of the QC sample, with percent recovery calculated as [(mean observed concentration)/(spiked concentration)] × 100%. Agilent MassHunter Work Station Software (B.10.00, Agilent Technologies) was used for MRM data acquisition and processing.

### Statistical analysis

The details of each experiment are provided in the figure legends. Data are expressed as the mean ± standard error of the mean (SEM). For data that conform to a normal distribution, a two-tailed unpaired Student’s *t*-test is used to determine statistical significance between two groups. For data that meet both normal distribution and homogeneity of variances, a one-way analysis of variance (ANOVA) and two-way ANOVA followed by the least significant difference (LSD) *post hoc* test are used to analyze multiple groups with only one variable tested and with multiple variables tested, respectively.^78,88^ For data that do not conform to normal distribution and/or homogeneity of variance, non-parametric tests are used to compare differences between groups. The Kruskal-Wallis and Wilcoxon rank sum tests were used to analyze the 16S rRNA gene sequencing and untargeted metabolomics data. A two-tailed *p* < 0.05 indicated statistical significance. All data were analyzed using IBM SPSS Statistics (version 20.0; SPSS Inc., Chicago, IL, USA).

## Supplementary Material

Supplemental Material

## Data Availability

The 16S sRNA gene sequencing and RNA sequencing raw files generated during this study are deposited into the NCBI Sequence Read Archive (SRA) database (http://www.ncbi.nlm.nih.gov/sra/) with the accession number PRJNA931923. Other data needed to evaluate the conclusions are present in the Supplementary Information: taxonomy annotations of fecal 16S rRNA gene sequencing in ND and HFD mice of different ages are presented in Additional file Data S1; taxonomy annotations of fecal 16S rRNA gene sequencing in the donor mice are presented in Additional file Data S2; RNA sequencing of the ovary tissues of FMT mice are attached in Additional file Data S3; untargeted metabolomics in the fecal samples of donor or FMT mice are included as Additional file Data S4; targeted metabolomics results for L-saccharopine and L-lysine in the serum, ovarian tissues, diets, and transmitted materials are presented in Additional file Data S5.

## References

[1] Laisk T, Tšuiko O, Jatsenko T, Hõrak P, Otala M, Lahdenperä M, Lummaa V, Tuuri T, Salumets A, Tapanainen JS. Demographic and evolutionary trends in ovarian function and aging. Hum Reprod Update. 2019;25(1):34–34. doi:10.1093/humupd/dmy031. 30346539

[2] Editorial. Recognizing the importance of ovarian aging research. Nat Aging. 2022;2(12):1071–1072. doi:10.1038/s43587-022-00339-0. 37118542

[3] Cavalcante MB, Sampaio OGM, Câmara FEA, Schneider A, de Ávila BM, Prosczek J, Masternak MM, Campos AR, de Ávila BM. Ovarian aging in humans: potential strategies for extending reproductive lifespan. Geroscience. 2023;45(4):2121–2133. doi:10.1007/s11357-023-00768-8. 36913129 PMC10651588

[4] Guida MC, Birse RT, Dall’agnese A, Toto PC, Diop SB, Mai A, Adams PD, Puri PL, Bodmer R. Intergenerational inheritance of high fat diet-induced cardiac lipotoxicity in drosophila. Nat Commun. 2019;10(1):193. doi:10.1038/s41467-018-08128-3. 30643137 PMC6331650

[5] Tong M, Saito T, Zhai P, Oka SI, Mizushima W, Nakamura M, Ikeda S, Shirakabe A, Sadoshima J. Mitophagy is essential for maintaining cardiac function during high fat diet-induced diabetic cardiomyopathy. Circ Res. 2019;124(9):1360–1371. doi:10.1161/CIRCRESAHA.118.314607. 30786833 PMC6483841

[6] Jiang J, Li Y, Liang S, Sun B, Shi Y, Xu Q, Zhang J, Shen H, Duan J, Sun Z. Combined exposure of fine particulate matter and high-fat diet aggravate the cardiac fibrosis in C57BL/6J mice. J Hazard Mater. 2020;391:122203. doi:10.1016/j.jhazmat.2020.122203. 32171159

[7] Evans AK, Saw NL, Woods CE, Vidano LM, Blumenfeld SE, Lam RK, Chu EK, Reading C, Shamloo M. Impact of high-fat diet on cognitive behavior and central and systemic inflammation with aging and sex differences in mice. Brain Behav Immun. 2024;118:334–354. doi:10.1016/j.bbi.2024.02.025. 38408498 PMC11019935

[8] Bu L, Zhang Z, Chen J, Fan Y, Guo J, Su Y, Wang H, Zhang X, Wu X, Jiang Q, et al. High-fat diet promotes liver tumorigenesis via palmitoylation and activation of AKT. Gut. 2024;73(7):1156–1168. doi:10.1136/gutjnl-2023-330826. 38191266

[9] Yuan C, Hong H, Wang N, Chen T, Cao M, Zhao Y, Shen C, Chen X, Luo Y, Zhang B, et al. Increased oxidized low-density lipoprotein in mice exposed to a high-fat diet impaired spermatogenesis by inhibiting testosterone synthesis via the Klk1bs/Eid3 pathway. Clin Transl Med. 2024;14(3):e1603. doi:10.1002/ctm2.1603. 38433441 PMC10909978

[10] Ding N, Zhang X, Zhang XD, Jing J, Liu SS, Mu YP, Peng LL, Yan YJ, Xiao GM, Bi XY, et al. Impairment of spermatogenesis and sperm motility by the high-fat diet-induced dysbiosis of gut microbes. Gut. 2020;69(9):1608–1619. doi:10.1136/gutjnl-2019-319127. 31900292 PMC7456731

[11] Di Berardino C, Peserico A, Capacchietti G, Zappacosta A, Bernabò N, Russo V, Mauro A, El Khatib M, Gonnella F, Konstantinidou F, et al. High-fat diet and female fertility across lifespan: a comparative lesson from mammal models. Nutrients. 2022;14(20):4341. doi:10.3390/nu14204341. 36297035 PMC9610022

[12] Chen X, Huang L, Cui L, Xiao Z, Xiong X, Chen C. Sodium-glucose cotransporter 2 inhibitor ameliorates high fat diet-induced hypothalamic-pituitary-ovarian axis disorders. J Physiol. 2022;600(21):4549–4568. doi:10.1113/JP283259. 36048516 PMC9826067

[13] Hohos NM, Elliott EM, Giornazi A, Silva E, Rice JD, Skaznik-Wikiel ME. High-fat diet induces an ovulatory defect associated with dysregulated endothelin-2 in mice. Reproduction. 2021;161(3):307–317. doi:10.1530/REP-20-0290. 33428588

[14] Ma X, Hayes E, Prizant H, Srivastava RK, Hammes SR, Sen A. Leptin-induced CART (cocaine- and amphetamine-regulated transcript) is a novel intraovarian Mediator of obesity-related infertility in females. Endocrinology. 2016;157(3):1248–1257. doi:10.1210/en.2015-1750. 26730935 PMC4769362

[15] Paula VG, Vesentini G, Sinzato YK, Moraes-Souza RQ, Volpato GT, Damasceno DC. Intergenerational high-fat diet impairs ovarian follicular development in rodents: a systematic review and meta-analysis. Nutr Rev. 2022;80(4):889–903. doi:10.1093/nutrit/nuab049. 34459492

[16] Adamowski M, Wołodko K, Oliveira J, Castillo-Fernandez J, Murta D, Kelsey G, Galvão AM. Leptin signaling in the ovary of diet-induced obese mice regulates activation of NOD-Like receptor protein 3 inflammasome. Front Cell Dev Biol. 2021;9:738731. doi:10.3389/fcell.2021.738731. 34805147 PMC8595835

[17] Hua D, Zhou Y, Lu Y, Zhao C, Qiu W, Chen J, Ju R. Lipotoxicity impairs granulosa cell function through activated endoplasmic reticulum stress pathway. Reprod Sci. 2020;27(1):119–131. doi:10.1007/s43032-019-00014-7. 32046379

[18] Clark KL, Roach CM, Keating AF. Obesity alters the ovarian DNA damage response and apoptotic proteins. Reproduction. 2020;160(5):751–760. doi:10.1530/REP-20-0070. 33021950

[19] Valdes AM, Walter J, Segal E, Spector TD. Role of the gut microbiota in nutrition and health. BMJ. 2018;361:k2179. doi:10.1136/bmj.k2179. 29899036 PMC6000740

[20] Lindell AE, Zimmermann-Kogadeeva M, Patil KR. Multimodal interactions of drugs, natural compounds and pollutants with the gut microbiota. Nat Rev Microbiol. 2022;20(7):431–443. doi:10.1038/s41579-022-00681-5. 35102308 PMC7615390

[21] Zhao L. The gut microbiota and obesity: from correlation to causality. Nat Rev Microbiol. 2013 Sep. 11(9):639–647. doi:10.1038/nrmicro3089. 23912213

[22] Rohr MW, Narasimhulu CA, Rudeski-Rohr TA, Parthasarathy S. Negative effects of a high-fat diet on intestinal permeability: a review. Adv Nutr. 2020;11(1):77–91. doi:10.1093/advances/nmz061. 31268137 PMC7442371

[23] Zhang XY, Chen J, Yi K, Peng L, Xie J, Gou X, Peng T, Tang L. Phlorizin ameliorates obesity-associated endotoxemia and insulin resistance in high-fat diet-fed mice by targeting the gut microbiota and intestinal barrier integrity. Gut Microbes. 2020;12(1):1–18. doi:10.1080/19490976.2020.1842990. PMC7714487 33222603

[24] Li S, Cai Y, Guan T, Zhang Y, Huang K, Zhang Z, Cao W, Guan X. Quinic acid alleviates high-fat diet-induced neuroinflammation by inhibiting DR3/IKK/NF-κB signaling via gut microbial tryptophan metabolites. Gut Microbes. 2024;16(1):2374608. doi:10.1080/19490976.2024.2374608. 38972055 PMC11229714

[25] Yuan J, Chen C, Cui J, Lu J, Yan C, Wei X, Zhao X, Li N, Li S, Xue G, et al. Fatty liver disease caused by High-Alcohol-Producing Klebsiella pneumoniae. Cell Metab. 2019;30(4):675–688.e7. doi:10.1016/j.cmet.2019.08.018. 31543403

[26] Qi X, Yun C, Sun L, Xia J, Wu Q, Wang Y, Wang L, Zhang Y, Liang X, Wang L, et al. Gut microbiota-bile acid-interleukin-22 axis orchestrates polycystic ovary syndrome. Nat Med. 2019;25(8):1225–1233. doi:10.1038/s41591-019-0509-0. 31332392 PMC7376369

[27] Huang F, Cao Y, Liang J, Tang R, Wu S, Zhang P, Chen R. The influence of the gut microbiome on ovarian aging. Gut Microbes. 2024;16(1):2295394. doi:10.1080/19490976.2023.2295394. 38170622 PMC10766396

[28] Penzias A, Azziz R, Bendikson K, Falcone T, Hansen K, Hill M, Hurd W, Jindal S, Kalra S, Mersereau J, et al. Practice committee of the American society for reproductive medicine. Testing and interpreting measures of ovarian reserve: a committee opinion. Fertil Steril. 2020;114(6):1151–1157. doi:10.1016/j.fertnstert.2020.09.134. 33280722

[29] Zhang H, Liu K. Cellular and molecular regulation of the activation of mammalian primordial follicles: somatic cells initiate follicle activation in adulthood. Hum Reprod Update. 2015;21(6):779–786. doi:10.1093/humupd/dmv037. 26231759

[30] Dong L, Teh DBL, Kennedy BK, Huang Z. Unraveling female reproductive senescence to enhance healthy longevity. Cell Res. 2023;33(1):11–29. doi:10.1038/s41422-022-00718-7. 36588114 PMC9810745

[31] Umehara T, Winstanley YE, Andreas E, Morimoto A, Williams EJ, Smith KM, Carroll J, Febbraio MA, Shimada M, Russell DL, et al. Female reproductive life span is extended by targeted removal of fibrotic collagen from the mouse ovary. Sci Adv. 2022;8(24):eabn4564. doi:10.1126/sciadv.abn4564. 35714185 PMC9205599

[32] Wei C, Li L, Menon MC, Zhang W, Fu J, Kidd B, Keung KL, Woytovich C, Greene I, Xiao W, et al. Genomic analysis of kidney allograft injury identifies hematopoietic cell kinase as a key Driver of renal fibrosis. J Am Soc Nephrol. 2017;28(5):1385–1393. doi:10.1681/ASN.2016020238. 27927780 PMC5407716

[33] Ye Q, Zeng X, Wang S, Zeng X, Yang G, Ye C, Cai S, Chen M, Li S, Qiao S. Butyrate drives the acetylation of histone H3K9 to activate steroidogenesis through PPARγ and PGC1α pathways in ovarian granulosa cells. FASEB J. 2021;35(2):e21316. doi:10.1096/fj.202000444R. 33433947

[34] Miller WL, Auchus RJ. The molecular biology, biochemistry, and physiology of human steroidogenesis and its disorders. Endocr Rev. 2011;32(1):81–151. doi:10.1210/er.2010-0013. 21051590 PMC3365799

[35] Di Lorenzo F, De Castro C, Silipo A, Molinaro A. Lipopolysaccharide structures of gram-negative populations in the gut microbiota and effects on host interactions. FEMS Microbiol Rev. 2019;43(3):257–272. doi:10.1093/femsre/fuz002. 30649292

[36] Matthews DE. Review of lysine metabolism with a focus on humans. J Nutr. 2020;150(Suppl 1):2548S–2555S. doi:10.1093/jn/nxaa224. 33000162

[37] Zhou J, Wang X, Wang M, Chang Y, Zhang F, Ban Z, Tang R, Gan Q, Wu S, Guo Y, et al. The lysine catabolite saccharopine impairs development by disrupting mitochondrial homeostasis. J Cell Biol. 2019;218(2):580–597. doi:10.1083/jcb.201807204. 30573525 PMC6363459

[38] May-Panloup P, Boucret L, Chao de la Barca JM, Desquiret-Dumas V, Ferré-L’Hotellier V, Morinière C, Descamps P, Procaccio V, Reynier P. Ovarian ageing: the role of mitochondria in oocytes and follicles. Hum Reprod Update. 2016;22(6):725–743. doi:10.1093/humupd/dmw028. 27562289

[39] Chiang JL, Shukla P, Pagidas K, Ahmed NS, Karri S, Gunn DD, Hurd WW, Singh KK. Mitochondria in ovarian aging and reproductive longevity. Ageing Res Rev. 2020;63:101168. doi:10.1016/j.arr.2020.101168. 32896666 PMC9375691

[40] Quintana-Cabrera R, Scorrano L. Determinants and outcomes of mitochondrial dynamics. Mol Cell. 2023;83(6):857–876. doi:10.1016/j.molcel.2023.02.012. 36889315

[41] Kraus F, Roy K, Pucadyil TJ, Ryan MT. Function and regulation of the divisome for mitochondrial fission. Nature. 2021;590(7844):57–66. doi:10.1038/s41586-021-03214-x. 33536648

[42] Valsangkar D, Downs SM. A requirement for fatty acid oxidation in the hormone-induced meiotic maturation of mouse oocytes. Biol Reprod. 2013;89(2):43. doi:10.1095/biolreprod.113.109058. 23863407 PMC4076365

[43] Miao Y, Cui Z, Gao Q, Rui R, Xiong B. Nicotinamide mononucleotide supplementation reverses the declining quality of maternally aged oocytes. Cell Rep. 2020;32(5):107987. doi:10.1016/j.celrep.2020.107987. 32755581

[44] Dalton CM, Carroll J. Biased inheritance of mitochondria during asymmetric cell division in the mouse oocyte. J Cell Sci. 2013;126(Pt 13):2955–2964. doi:10.1242/jcs.128744. 23659999 PMC3699109

[45] He H, Wang J, Mou X, Liu X, Li Q, Zhong M, Luo B, Yu Z, Zhang J, Xu T, et al. Selective autophagic degradation of ACLY (ATP citrate lyase) maintains citrate homeostasis and promotes oocyte maturation. Autophagy. 2023;19(1):163–179. doi:10.1080/15548627.2022.2063005. 35404187 PMC9809967

[46] Huang BB, Liu XC, Qin XY, Chen J, Ren PG, Deng WF, Zhang J. Effect of high-fat diet on immature female mice and Messenger and noncoding RNA expression profiling in ovary and white adipose tissue. Reprod Sci. 2019;26(10):1360–1372. doi:10.1177/1933719118765966. 29642802

[47] Salinero AE, Anderson BM, Zuloaga KL. Sex differences in the metabolic effects of diet-induced obesity vary by age of onset. Int J Obes (Lond). 2018;42(5):1088–1091. doi:10.1038/s41366-018-0023-3. 29463918

[48] Becerril S, Rodríguez A, Catalán V, Ramírez B, Mentxaka A, Neira G, Gómez-Ambrosi J, Frühbeck G. Sex- and age-dependent changes in the Adiponectin/Leptin ratio in experimental Diet-induced obesity in mice. Nutrients. 2022;15(1):73. doi:10.3390/nu15010073. 36615734 PMC9823624

[49] Aurich AC, Niemann B, Pan R, Gruenler S, Issa H, Silber RE, Rohrbach S. Age-dependent effects of high fat-diet on murine left ventricles: role of palmitate. Basic Res Cardiol. 2013;108(5):369. doi:10.1007/s00395-013-0369-6. 23836256

[50] Sakamoto Y, Niwa M, Muramatsu K, Shimo S. High-fat diet and age-dependent effects of IgA-bearing cell populations in the small intestinal lamina propria in mice. Int J Mol Sci. 2021;22(3):1165. doi:10.3390/ijms22031165. 33503874 PMC7866202

[51] Zhang T, Sun P, Geng Q, Fan H, Gong Y, Hu Y, Shan L, Sun Y, Shen W, Zhou Y. Disrupted spermatogenesis in a metabolic syndrome model: the role of vitamin a metabolism in the gut-testis axis. Gut. 2022;71(1):78–87. doi:10.1136/gutjnl-2020-323347. 33504491 PMC8666830

[52] Fan Z, Zhang X, Shang Y, Zou M, Zhou M, Q E, Fei S, Chen W, Li J, Zhang X, et al. Intestinal flora changes induced by a high-fat diet promote activation of primordial follicles through macrophage infiltration and inflammatory factor secretion in Mouse Ovaries. Int J Mol Sci. 2022;23(9):4797. doi:10.3390/ijms23094797. 35563189 PMC9100959

[53] Pan RY, Zhang J, Wang J, Wang Y, Li Z, Liao Y, Liao Y, Zhang C, Liu Z, Song L, et al. Intermittent fasting protects against Alzheimer’s disease in mice by altering metabolism through remodeling of the gut microbiota. Nat Aging. 2022;2(11):1024–1039. doi:10.1038/s43587-022-00311-y. 37118092

[54] de Vos WM, Tilg H, Van Hul M, Cani PD. Gut microbiome and health: mechanistic insights. Gut. 2022;71(5):1020–1032. doi:10.1136/gutjnl-2021-326789. 35105664 PMC8995832

[55] Zhao WP, Wang HW, Liu J, Zhang ZH, Zhu SQ, Zhou BH. Mitochondrial respiratory chain complex abnormal expressions and fusion disorder are involved in fluoride-induced mitochondrial dysfunction in ovarian granulosa cells. Chemosphere. 2019;215:619–625. doi:10.1016/j.chemosphere.2018.10.043. 30342406

[56] Zhang X, Wu XQ, Lu S, Guo YL, Ma X. Deficit of mitochondria-derived ATP during oxidative stress impairs mouse MII oocyte spindles. Cell Res. 2006;16(10):841–850. doi:10.1038/sj.cr.7310095. 16983401

[57] Kirillova A, Smitz JEJ, Sukhikh GT, Mazunin I. The role of mitochondria in oocyte maturation. Cells. 2021;10(9):2484. doi:10.3390/cells10092484. 34572133 PMC8469615

[58] van der Reest J, Nardini Cecchino G, Haigis MC, Kordowitzki P. Mitochondria: their relevance during oocyte ageing. Ageing Res Rev. 2021;70:101378. doi:10.1016/j.arr.2021.101378. 34091076

[59] Zhang H, Pan Z, Ju J, Xing C, Li X, Shan M, Sun S. DRP1 deficiency induces mitochondrial dysfunction and oxidative stress-mediated apoptosis during porcine oocyte maturation. J Anim Sci Biotechnol. 2020;11(1):77. doi:10.1186/s40104-020-00489-4. 32782788 PMC7409671

[60] Tamura H, Takasaki A, Miwa I, Taniguchi K, Maekawa R, Asada H, Taketani T, Matsuoka A, Yamagata Y, Shimamura K, et al. Oxidative stress impairs oocyte quality and melatonin protects oocytes from free radical damage and improves fertilization rate. J Pineal Res. 2008;44(3):280–287. doi:10.1111/j.1600-079X.2007.00524.x. 18339123

[61] Greaney J, Wei Z, Homer H. Regulation of chromosome segregation in oocytes and the cellular basis for female meiotic errors. Hum Reprod Update. 2018;24(2):135–161. doi:10.1093/humupd/dmx035. 29244163

[62] Xu Y, Sun MH, Xu Y, Ju JQ, Pan MH, Pan ZN, Li XH, Sun SC. Nonylphenol exposure affects mouse oocyte quality by inducing spindle defects and mitochondria dysfunction. Environ Pollut. 2020;266(Pt 1):114967. doi:10.1016/j.envpol.2020.114967. 32645552

[63] Dumont J, Desai A. Acentrosomal spindle assembly and chromosome segregation during oocyte meiosis. Trends Cell Biol. 2012;22(5):241–249. doi:10.1016/j.tcb.2012.02.007. 22480579 PMC3348331

[64] Cerf-Bensussan N, Gaboriau-Routhiau V. The immune system and the gut microbiota: friends or foes? Nat Rev Immunol. 2010;10(10):735–744. doi:10.1038/nri2850. 20865020

[65] Kamada N, Seo SU, Chen GY, Núñez G. Role of the gut microbiota in immunity and inflammatory disease. Nat Rev Immunol. 2013;13(5):321–335. doi:10.1038/nri3430. 23618829

[66] Alloo J, Leleu I, Grangette C, Pied S. Parasite infections, neuroinflammation, and potential contributions of gut microbiota. Front Immunol. 2022;13:1024998. doi:10.3389/fimmu.2022.1024998. 36569929 PMC9772015

[67] Hasan RA, Koh AY, Zia A. The gut microbiome and thromboembolism. Thromb Res. 2020;189:77–87. doi:10.1016/j.thromres.2020.03.003. 32192995 PMC8780211

[68] Pawlinski R, Wang JG, Owens AP 3rd, Williams J, Antoniak S, Tencati M, Luther T, Rowley JW, Low EN, Weyrich AS, et al. Hematopoietic and nonhematopoietic cell tissue factor activates the coagulation cascade in endotoxemic mice. Blood. 2010;116(5):806–814. doi:10.1182/blood-2009-12-259267. 20410508 PMC2918334

[69] Yang X, Cheng X, Tang Y, Qiu X, Wang Y, Kang H, Wu J, Wang Z, Liu Y, Chen F, et al. Bacterial endotoxin activates the coagulation cascade through gasdermin D-Dependent phosphatidylserine exposure. Immunity. 2019;51(6):983–996.e6. doi:10.1016/j.immuni.2019.11.005. 31836429

[70] Blüher M. Obesity: global epidemiology and pathogenesis. Nat Rev Endocrinol. 2019;15(5):288–298. doi:10.1038/s41574-019-0176-8. 30814686

[71] Freeman EW, Gracia CR, Sammel MD, Lin H, Lim LC, Strauss JF 3rd. Association of anti-mullerian hormone levels with obesity in late reproductive-age women. Fertil Steril. 2007;87(1):101–106. doi:10.1016/j.fertnstert.2006.05.074. 17109858

[72] He Y, Tian J, Oddy WH, Dwyer T, Venn AJ. Association of childhood obesity with female infertility in adulthood: a 25-year follow-up study. Fertil Steril. 2018;110(4):596–604.e1. doi:10.1016/j.fertnstert.2018.05.011. 30196944

[73] Maheshwari A, Stofberg L, Bhattacharya S. Effect of overweight and obesity on assisted reproductive technology–a systematic review. Hum Reprod Update. 2007;13(5):433–444. doi:10.1093/humupd/dmm017. 17584821

[74] Metwally M, Ong KJ, Ledger WL, Li TC. Does high body mass index increase the risk of miscarriage after spontaneous and assisted conception? A meta-analysis of the evidence. Fertil Steril. 2008;90(3):714–726. doi:10.1016/j.fertnstert.2007.07.1290. 18068166

[75] Neshich IA, Kiyota E, Arruda P. Genome-wide analysis of lysine catabolism in bacteria reveals new connections with osmotic stress resistance. Isme J. 2013;7(12):2400–2410. doi:10.1038/ismej.2013.123. 23887172 PMC3834855

[76] Wu L, Meng J, Shen Q, Zhang Y, Pan S, Chen Z, Zhu LQ, Lu Y, Huang Y, Zhang G. Caffeine inhibits hypothalamic A1R to excite oxytocin neuron and ameliorate dietary obesity in mice. Nat Commun. 2017;8(1):15904. doi:10.1038/ncomms15904. 28654087 PMC5490268

[77] Hong Y, Lin Y, Si Q, Yang L, Dong W, Gu X. Ginsenoside Rb2 alleviates obesity by activation of Brown Fat and induction of Browning of white Fat. Front Endocrinol (Lausanne). 2019;10:153. doi:10.3389/fendo.2019.00153. 30930854 PMC6428988

[78] Luo W, Ye L, Hu XT, Wang MH, Wang MX, Jin LM, Xiao ZX, Qian JC, Wang Y, Zuo W, et al. MD2 deficiency prevents high-fat diet-induced AMPK suppression and lipid accumulation through regulating TBK1 in non-alcoholic fatty liver disease. Clin Transl Med. 2022 Mar. 12(3):e777. doi:10.1002/ctm2.777. 35343085 PMC8958353

[79] Hansen AK, Krych Ł, Nielsen DS, Hansen CH. A review of applied aspects of dealing with gut microbiota impact on rodent models. Ilar J. 2015;56(2):250–264. doi:10.1093/ilar/ilv010. 26323634

[80] Wernstedt Asterholm I, Tao C, Morley TS, Wang QA, Delgado-Lopez F, Wang ZV, Scherer PE. Adipocyte inflammation is essential for healthy adipose tissue expansion and remodeling. Cell Metab. 2014;20(1):103–118. doi:10.1016/j.cmet.2014.05.005. 24930973 PMC4079756

[81] Byers SL, Wiles MV, Dunn SL, Taft RA, Singh SR. Mouse estrous cycle identification tool and images. PLoS One. 2012;7(4):e35538. doi:10.1371/journal.pone.0035538. 22514749 PMC3325956

[82] Gao Y, Wu T, Tang X, Wen J, Zhang Y, Zhang J, Wang S. Increased cellular senescence in doxorubicin-induced murine ovarian injury: effect of senolytics. Geroscience. 2023;45(3):1775–1790. doi:10.1007/s11357-023-00728-2. 36648735 PMC10400526

[83] Ajayi AF, Akhigbe RE. Staging of the estrous cycle and induction of estrus in experimental rodents: an update. Fertil Res Pract. 2020;6(1):5. doi:10.1186/s40738-020-00074-3. 32190339 PMC7071652

[84] Suwandhi L, Altun I, Karlina R, Miok V, Wiedemann T, Fischer D, Walzthoeni T, Lindner C, Böttcher A, Heinzmann SS, et al. Asc-1 regulates white versus beige adipocyte fate in a subcutaneous stromal cell population. Nat Commun. 2021;12(1):1588. doi:10.1038/s41467-021-21826-9. 33707431 PMC7952576

[85] Zhou S, Xi Y, Chen Y, Zhang Z, Wu C, Yan W, Luo A, Wu T, Zhang J, Wu M, et al. Ovarian dysfunction induced by chronic whole-body PM2.5 exposure. Small. 2020;16(33):e2000845. doi:10.1002/smll.202000845. 32686359

[86] Tian Y, Shen W, Lai Z, Shi L, Yang S, Ding T, Wang S, Luo A. Isolation and identification of ovarian theca-interstitial cells and granulose cells of immature female mice. Cell Biol Int. 2015;39(5):584–590. doi:10.1002/cbin.10426. 25640196

[87] Yuan S, Wen J, Cheng J, Shen W, Zhou S, Yan W, Shen L, Luo A, Wang S. Age-associated up-regulation of EGR1 promotes granulosa cell apoptosis during follicle atresia in mice through the nf-κB pathway. Cell Cycle. 2016;15(21):2895–2905. doi:10.1080/15384101.2016.1208873. 27436181 PMC5105915

[88] Song W, Wang Y, Li G, Xue S, Zhang G, Dang Y, Wang H. Modulating the gut microbiota is involved in the effect of low-molecular-weight Glycyrrhiza polysaccharide on immune function. Gut Microbes. 2023;15(2):2276814. doi:10.1080/19490976.2023.2276814. 37948152 PMC10653635

