# Supplementary material for: High-fat diet-induced L-saccharopine accumulation inhibits estradiol synthesis and damages oocyte quality by disturbing mitochondrial homeostasis
Source: Gut Microbes. 2024 Oct 16;16(1):2412381. doi: 10.1080/19490976.2024.2412381 (PMC11485700; doi:10.1080/19490976.2024.2412381)
Supplement: Supplemental Material [file KGMI_A_2412381_SM5753.zip › KGMI_A_2412381 (1)/Additional file 1.docx]

# Supplementary figures


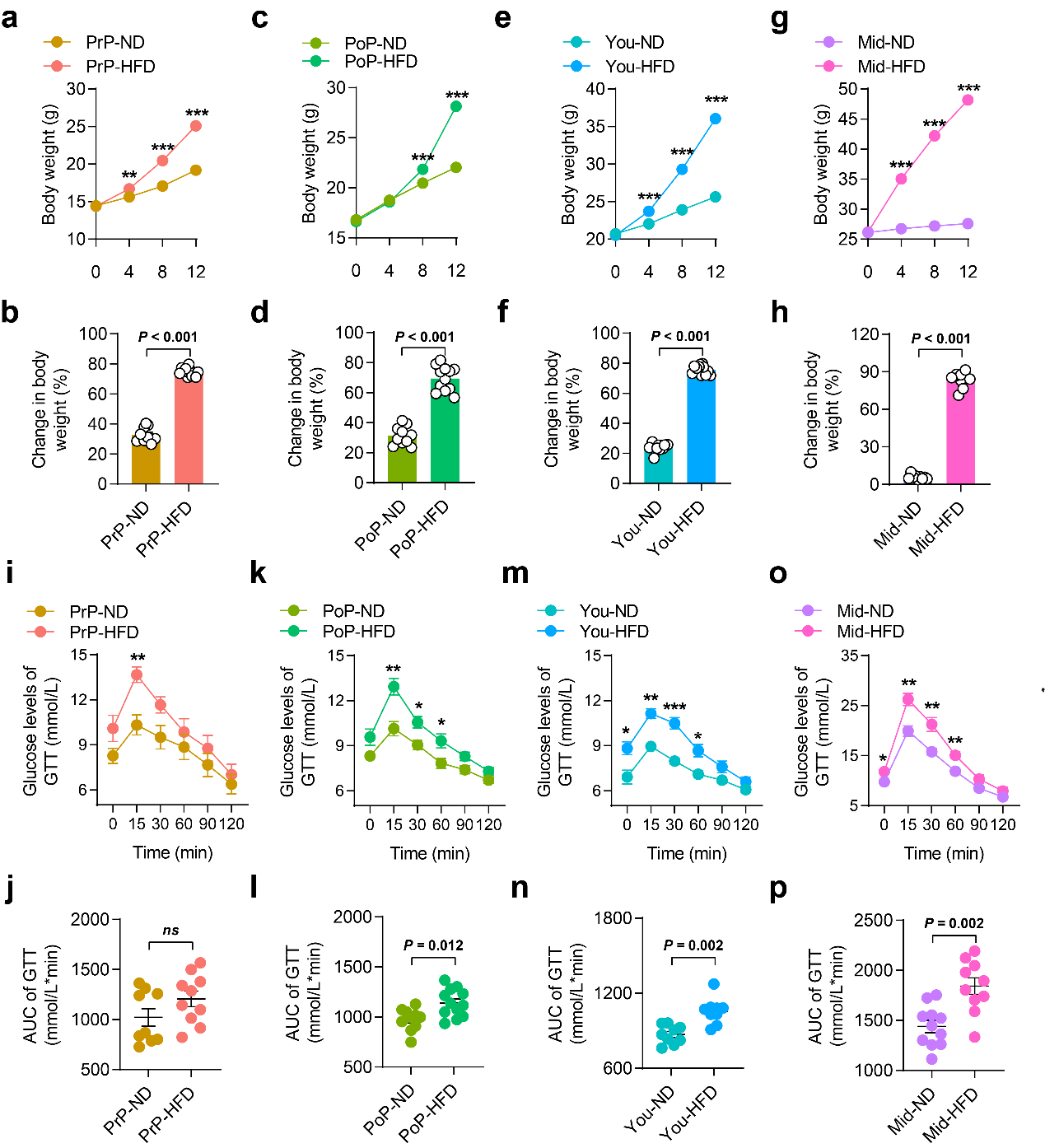


**Fig. S1 HFD induced metabolic disorders at different ages. a-h**, Change in body weights over 12 weeks (*n* = 9-12). **i-p**, Glucose tolerance test (GTT) with area under the curve (AUC) (*n* = 9-12). Individual values are displayed as dots, while mean ± SEM is shown as a column and error bar. Statistical significance was determined by The Kruskal - Wallis rank sum test. *P* < 0.05 was considered statistically significant. ^*^*P* < 0.05, ^**^*P* < 0.01, ^***^*P* < 0.001. ND, normal diet; HFD, high-fat diet; PrP, pre-puberty; PoP, post-puberty; You, young adult; Mid, middle age.


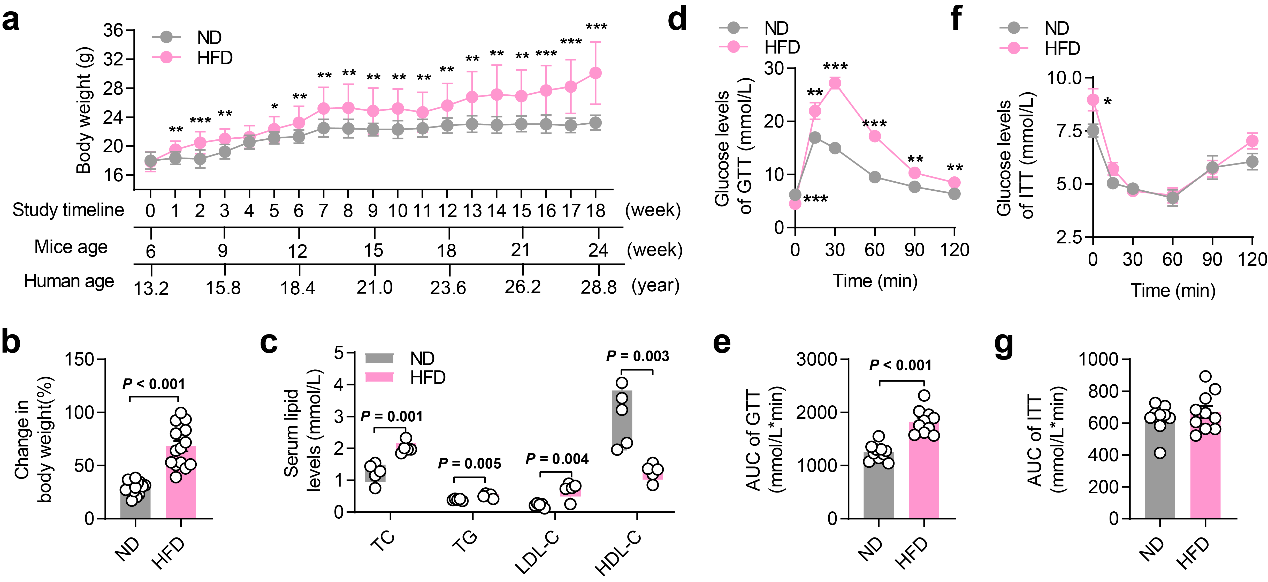


**Fig. S2 HFD induced metabolic disorders in the donor mice. a**, **b**, Change in body weights over 18 weeks (*n* = 15). **c**, Comparison of the serum TC, TG, LDL-C, and HDL-C levels between the ND and HFD group (*n* = 5). **d**, **e**, Glucose tolerance test (GTT) with area under the curve (AUC) (*n* = 10). **f**, **g**, Insulin tolerance test (ITT) with AUC (*n* = 10). Individual values are displayed as dots, while mean ± SEM is shown as a column and error bar. Statistical significance was determined by a two-tailed unpaired Student’s *t*-test. *P* < 0.05 was considered statistically significant. ^*^*P* < 0.05, ^**^*P* < 0.01, ^***^*P* < 0.001. ND, normal diet; HFD, high-fat diet; TC, total cholesterol; TG, total triglyceride; LDL-C, low density lipoprotein- cholesterol; HDL-C, high density lipoprotein- cholesterol.

**
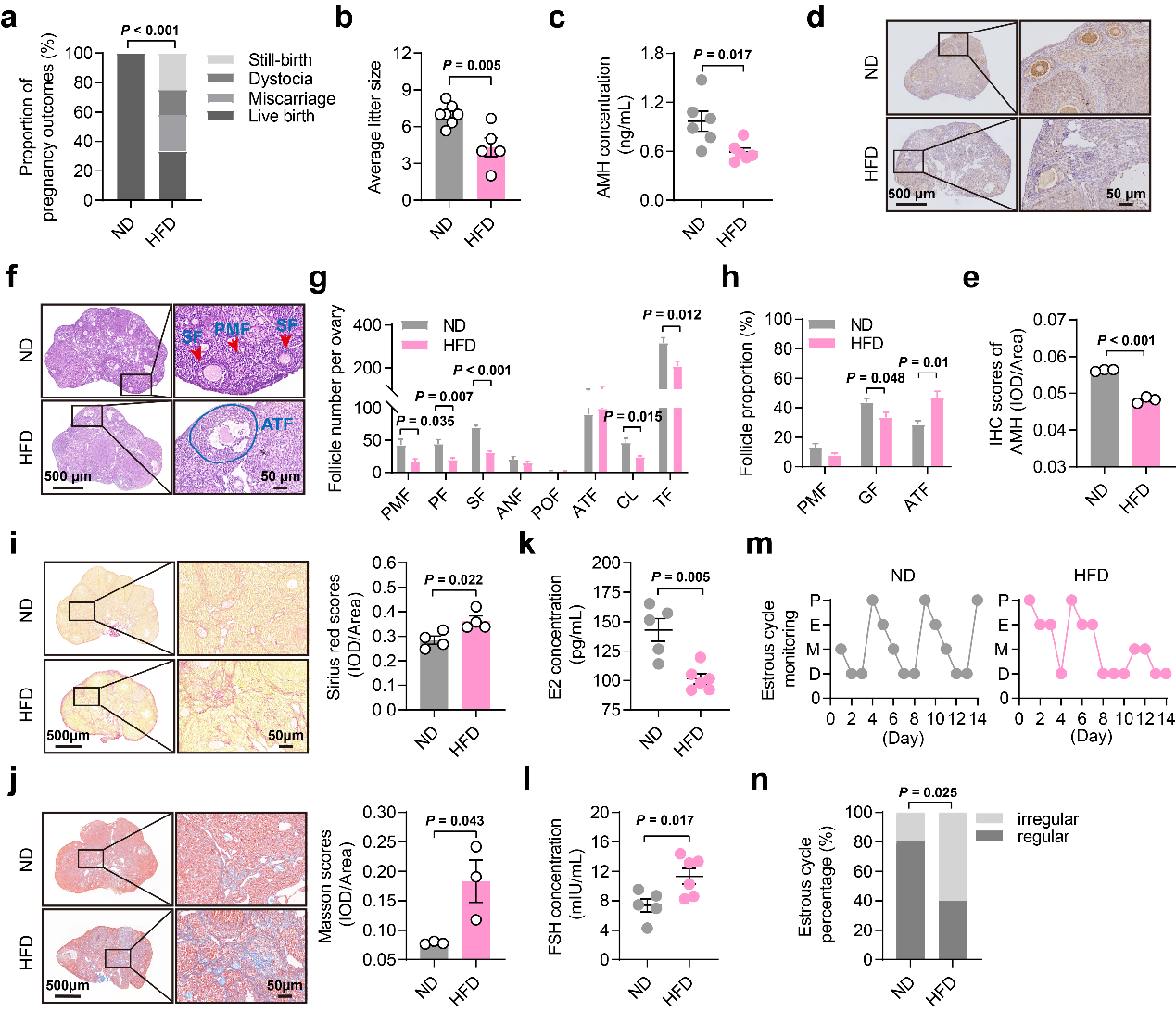
**

**Fig. S3 HFD induced ovarian dysfunction in the donor mice. a**, Comparison of pregnancy outcomes between the ND and HFD group. **b**, Comparison of average litter size between the ND and HFD group (*n* ≥ 5). **c**, Serum AMH levels in mice (*n* ≥ 5). **d, e**, Protein expression of AMH in the ovary of mice by immunohistochemistry (*n* = 3). Scale bars, 500 μm and 50 μm. **f**, Representative H&E staining images of mice ovaries. Scale bar, 500 μm and 50 μm. **g**, Follicle counts according to ovarian serial sections (*n* = 5). **h**, Proportion of follicles at different stages (*n* = 5). **i**, Representative images for Sirius Red staining of ovaries and Sirius Red staining scores of ovaries based on IOD/Area (*n* = 4). Scale bars, 500 μm and 50 μm. **j**, Representative images for Masson staining of ovaries and Masson staining scores of ovaries based on IOD/Area (*n* = 3). Scale bars, 500 μm and 50 μm. **k**, Serum E2 levels in mice (*n* ≥ 5). **l**, Serum FSH levels in mice (*n* ≥ 5). **m**, Representative estrous cycles. P, proestrus; E, estrus; M, metestrus; D, diestrus. **n**, Percentage of irregular estrous cycles (*n* = 15). Individual values are displayed as dots, while mean ± SEM is shown as a column and error bar. Statistical significance was determined by Chi-square test (**a, n**), or a two-tailed unpaired Student’s *t*-test. *P* < 0.05 was considered statistically significant. ^*^*P* < 0.05, ^**^*P* < 0.01, ^***^*P* < 0.001. ND, normal diet; HFD, high-fat diet; AMH, anti-müllerian hormone; PMF, primordial follicle; PF, primary follicle; SF, secondary follicle; ANF, antral follicle; POF, pre-ovulatory follicle; ATF, atretic follicle; CL, corpus luteum; TF, total follicle; GF, growing follicle; E2, estradiol; FSH, follicle-stimulating hormone.

**
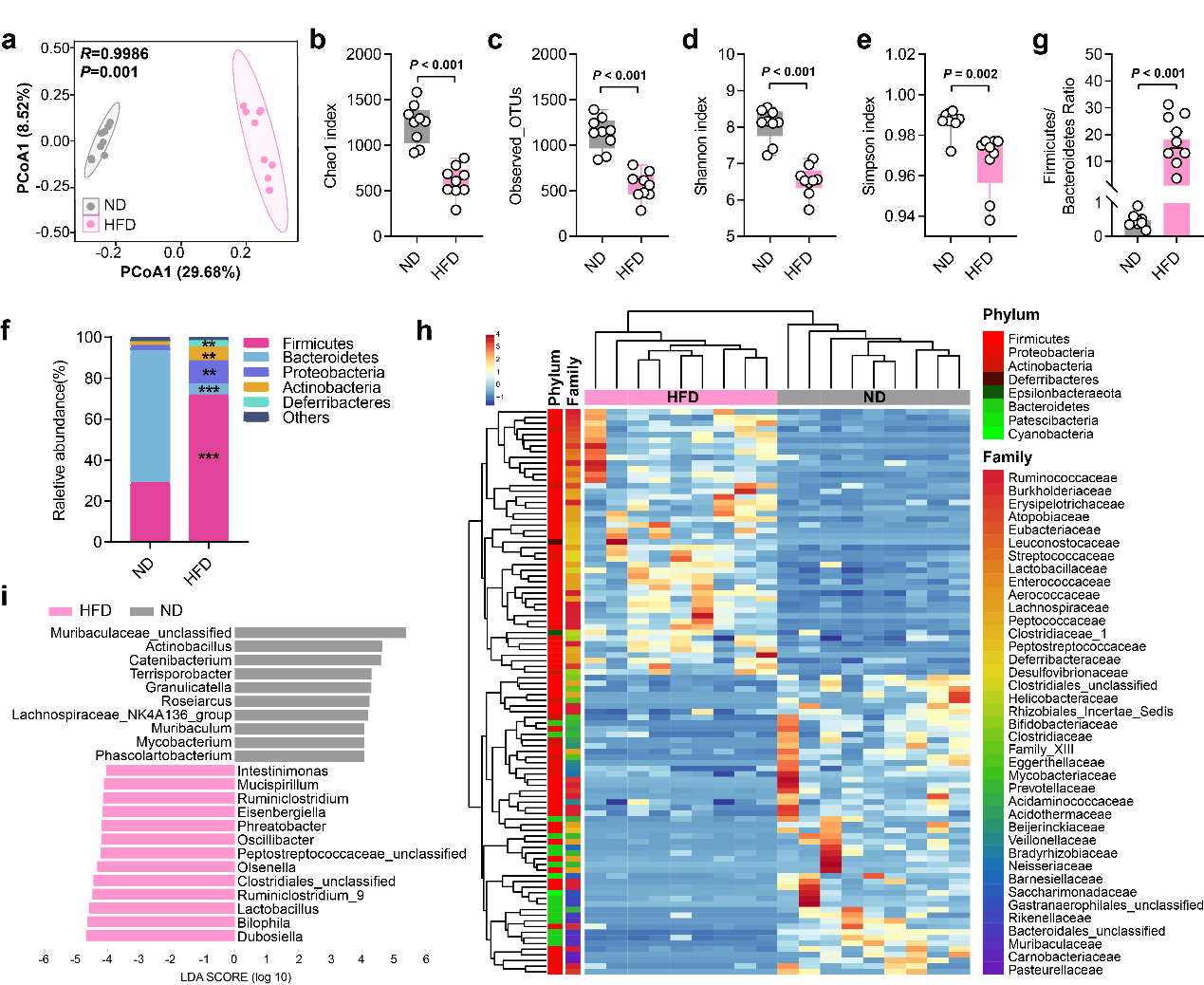
**

**Fig. S4 HFD induced gut microbiota dysbiosis in the donor mice.** **a**, PCoA plot of gut microbiota in the ND and HFD group (*n* = 9). **b-e**, Alpha indices of gut microbiota in mice (*n* = 9). **f**, Relative abundance of microbiota at the phylum level in the two groups (*n* = 9). **g**, Ratio of the relative abundance of *Firmicutes* and *Bacteroidetes* in the gut (*n* = 9). **h**, Differentially abundant bacteria between the ND and the HFD group. This idealized tree represents the taxonomic hierarchy down to the genus level with bars that are color-coded for phylum and family (*n* = 9). **i**, Histogram of LDA scores for differentially abundant bacterial genera (LDA > 4) (*n* = 9). Individual values are displayed as dots, while mean ± SEM. is shown as a column and error bar. Statistical significance was determined by Wilcoxon rank-sum test. *P* < 0.05 was considered statistically significant. ^**^*P* < 0.01, ^***^*P* < 0.001. ND, normal diet; HFD, high-fat diet; PCoA, principal coordinates analysis; LDA, linear discriminant analysis.


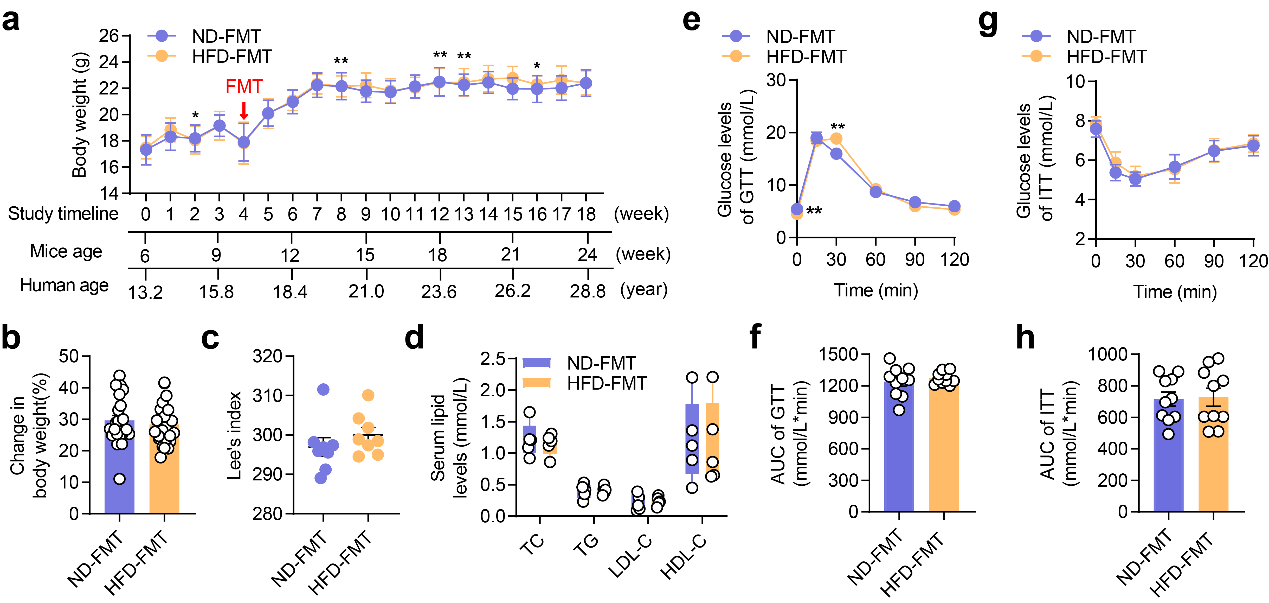


**Fig. S5 Fecal microbiota transplanted from HFD mice did not induce metabolic disorder in recipient mice. a**, **b**, Change in body weights over a period of 18 weeks (*n* = 25). **c**, Comparison of Lee’s index (*n* = 8). **d**, Comparison of serum TC, TG, LDL-C, and HDL-C levels (*n* = 5). **e**, **f**, Glucose tolerance test (GTT) with area under the curve (AUC) (*n* = 10). **g**, **h**, Insulin tolerance test (ITT) with AUC (*n* = 10). Individual values are displayed as dots, while mean ±SEM is shown as a column and error bar. Statistical significance was determined by a two-tailed unpaired Student’s *t*-test. *P* < 0.05 was considered statistically significant. ^*^*P* < 0.05, ^**^*P* < 0.01. ND, normal diet; HFD, high-fat diet; FMT, fecal microbiota transplantation; TC, total cholesterol; TG, total triglyceride; LDL-C, low density lipoprotein- cholesterol; HDL-C, high density lipoprotein- cholesterol.


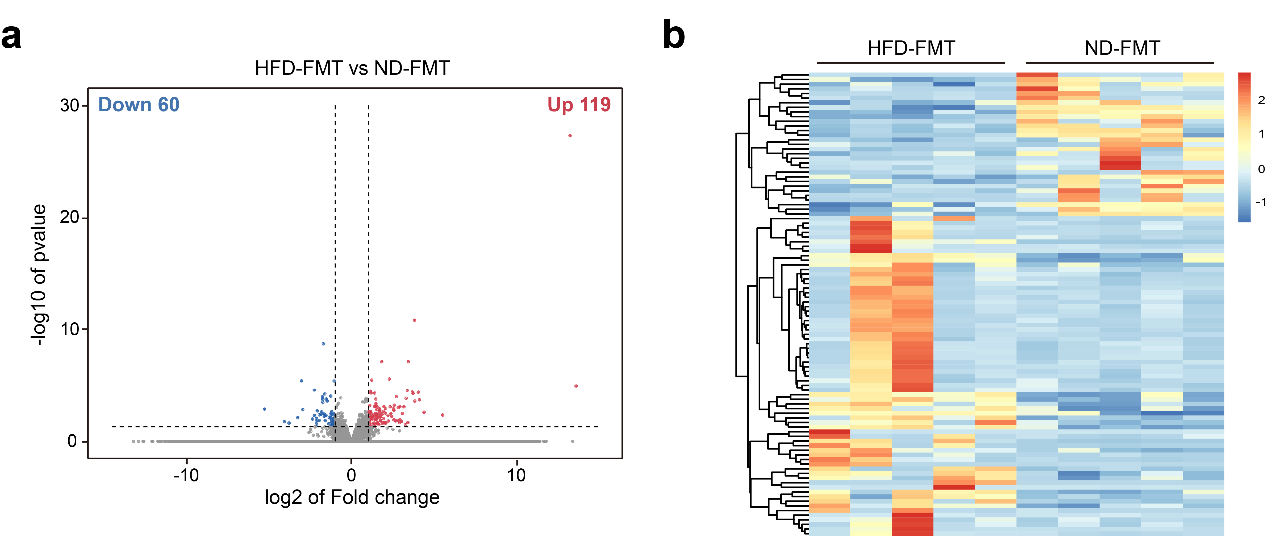


**Fig. S6 Fecal microbiota transplanted from HFD mice altered the gene expression in the ovaries of recipient mice. a**, Volcano plot of genes. Red and blue dots indicate the upregulated and downregulated genes in the HFD-FMT group, respectively (*n* = 5). **b**, Heat map of the DEGs (*n* = 5). ND, normal diet; HFD, high-fat diet; FMT, fecal microbiota transplantation; DEGs, differentially expressed genes.


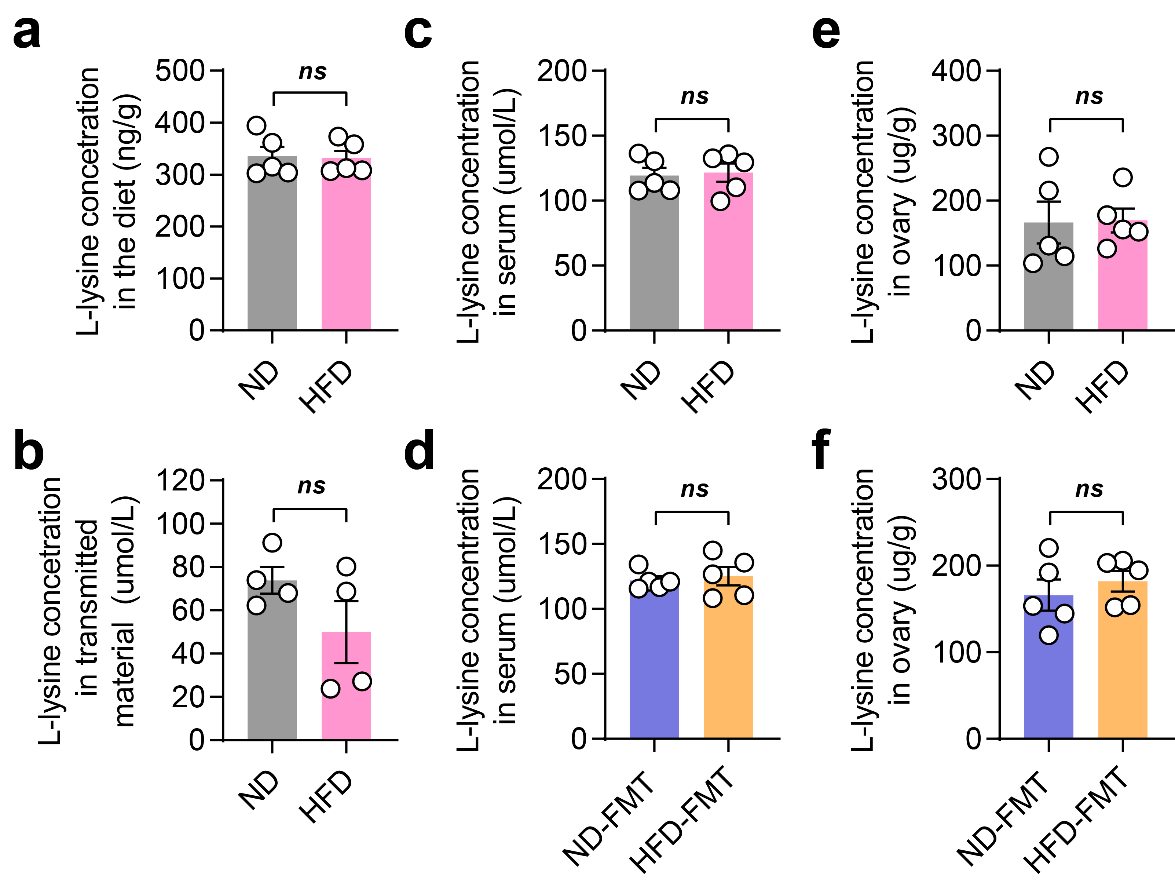


**Fig. S7 Concentration analysis of L-lysine by UHPLC-MS/MS. a, b**, Concentration analysis of L-lysine in the diet (*n* = 5) and transmitted material (*n* = 4) from the ND and HFD groups. **c-f**, Serum and ovarian L-lysine levels of the ND, HFD, ND-FMT, and HFD-FMT mice (*n* = 5). ND, normal diet; HFD, high-fat diet; *ns*, no significant difference; FMT, fecal microbiota transplantation; UHPLC, ultra-high performance liquid chromatography; MS, mass spectrometry.


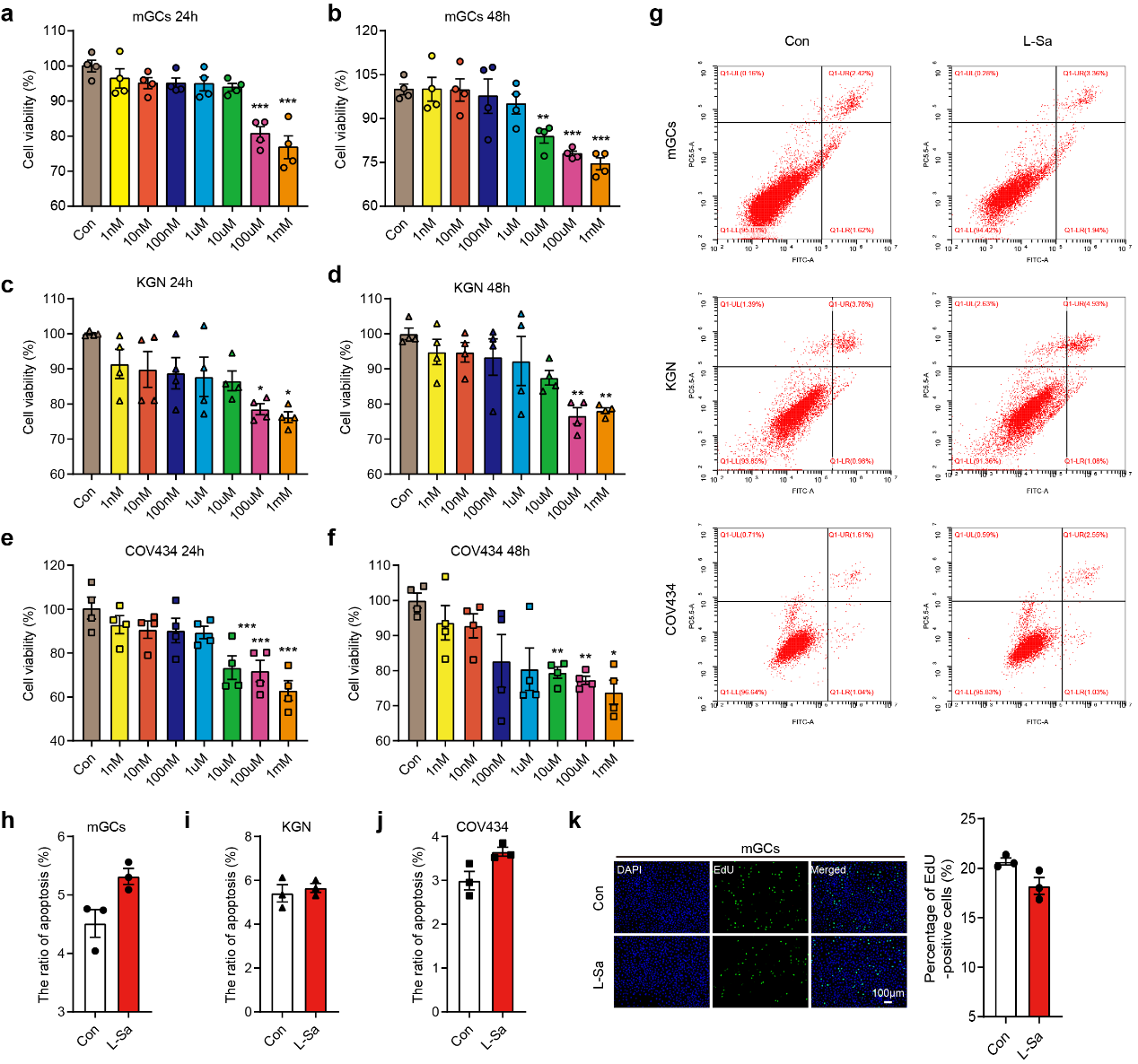


**Fig. S8** **Effects of L-saccharopine on cell viability, proliferation and apoptotic capacity. a-f**, Measurement of cell viability upon L-Sa treatment for 24 and 48 h with CCK8 kit (*n* = 4). **g-j**, Apoptosis rate of cells treated with L-Sa (100nM) for 48 h, determined using flow cytometry (*n* = 3). **k**, Proliferation of mGCs in the control and L-Sa (100nM) groups measured using the EdU incorporation assay (*n* = 3). Scale bar, 100 μm. Individual values are displayed as dots, while mean ± SEM is shown as a column and error bar. Statistical significance was determined by one-way ANOVA followed by LSD multiple comparisons test (**a-f**) or a two-tailed unpaired Student’s *t*-test (**h-k**). *P* < 0.05 was considered statistically significant. L-Sa, L-saccharopine; mGCs, mouse granulosa cells; KGN, COV434, human granulosa-like tumor cell lines.
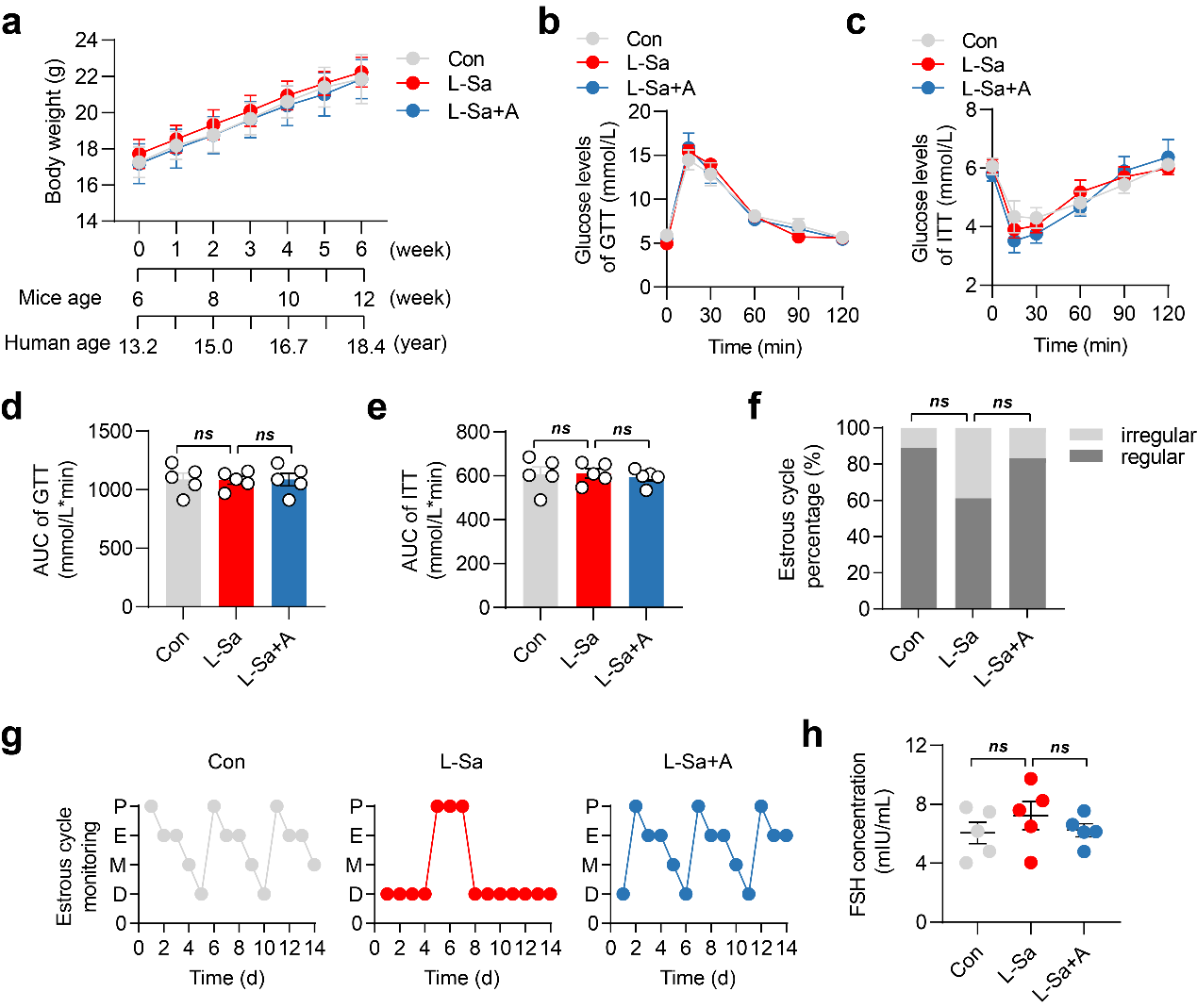


**Fig. S9 L-saccharopine did not affect the body weight and glucose metabolism in mice. a**, Change in body weights over a period of 6 weeks (*n* = 18). **b**, **d**, Glucose tolerance test (GTT) with area under the curve (AUC) (*n* = 5). **c**, **e**, Insulin tolerance test (ITT) with AUC (*n* = 5). **f**, Percentage of irregular estrous cycles (*n* = 18). **g**, Representative estrous cycles. P, proestrus; E, estrus; M, metestrus; D, diestrus. **h**, Serum FSH concentrations in mice (*n* = 5). Individual values are displayed as dots, while mean ±SEM is shown as a column and error bar. Statistical significance was determined by a one-way ANOVA, followed by LSD multiple comparisons test. *P* < 0.05 was considered statistically significant. Con, control; L-Sa, L-saccharopine; A, Acadesine (AICR); GTT, glucose tolerance test; AUC, area under the curve; ITT, insulin tolerance test; *ns*, no significant difference; FSH, follicle-stimulating hormone.

**Table S1. Detail description of 60% high-fat diet (MD12033) and normal diet (MD12031).**

**Caloric information (by kcal%).**

| Nutrient/diets | ND (MD12031, kcal%) | HFD (MD12033, kcal%) |
| --- | --- | --- |
| Protein | 20 | 20 |
| Fat | 10 | 60 |
| Carbohydrate | 70 | 20 |

ND, normal diet; HFD, high-fat diet; kcal%, kilocalorie percent.

**Detail description of** **60% high-fat diet (MD12033).**

| Class description | Ingredient | %100g |
| --- | --- | --- |
| Protein | Casein | 26.76% |
| Carbohydrate | Corn starch | 0 |
| Carbohydrate | Maltodextrin | 16.15 |
| Carbohydrate | Saccharose | 8.89 |
| Fiber | Solka Floc | 6.46 |
| Fat | Soybean oil | 3.23 |
| Fat | Lard | 31.66 |
| Mineral  Vitamin  Vitamin  Dye | S10026B  V10001C  Choline bitartrate  Dye Blue FD&C #1, Alum. Lake 35-42% | 6.46  0.13  0.26  ＜0.01 |

**Composition of normal diet (MD12031).**

| Class description | Ingredient | %100g |
| --- | --- | --- |
| Protein | Casein | 18.96 |
| Carbohydrate | Corn starch | 41.69 |
| Carbohydrate | Maltodextrin | 7.11 |
| Carbohydrate | Saccharose | 16.38 |
| Fiber | Solka Floc | 4.74 |
| Fat | Soybean oil | 2.37 |
| Fat | Lard | 1.90 |
| Mineral  Vitamin  Vitamin | S10026B  V10001C  Choline bitartrate | 6.46  0.13  0.26 |

**Table S2. The detection range, sensitivity, intra-assay and inter-assay precision of ELISA kits used in our studies.**

| ELISA Kit | Manufacturer | Catalog Number | Detection Range | Sensitivity | Intra-assay Precision | Inter-assay Precision |
| --- | --- | --- | --- | --- | --- | --- |
| Mouse E2 ELISA Kit | Cusabio Technology | CSB-E05109m | 40-1000 pg/mL | 40 pg/mL | <15% | <15% |
| Mouse FSH ELISA Kit | Cusabio Technology | CSB-E06871m | 4-140 mIU/mL | 2.5 mIU/mL | <15% | <15% |
| Mouse AMH ELISA kit | Cusabio Technology | CSB-E13156m | 0.4-14 ng/mL | 0.25 ng/mL | <15% | <15% |
| Mouse LPS ELISA Kit | Cusabio Technology | CSB-E13066m | 0.156-10 ng/mL | 0.039 ng/mL | <8% | <10% |
| Mouse TNF-α ELISA Kit | Cusabio Technology | CSB-E04741m | 62.5-4000 pg/mL | 15.6 pg/mL | <8% | <10% |
| Mouse IL-1β ELISA Kit | Cusabio Technology | CSB-E08054m | 31.25-2000 pg/mL | 7.8 pg/mL | <8% | <10% |
| Mouse IL-6 ELISA Kit | Cusabio Technology | CSB-E04639m | 1.56-100 pg/mL | 0.39 pg/mL | <8% | <10% |
| E2 ELISA kit | Cloud-Clone Corp | CEA461Ge | 12.35-1000 pg/mL | 4.45 pg/mL | <10% | <12% |

ELISA, enzyme-linked immunosorbent assay; E2, estradiol; FSH, follicle-stimulating hormone, AMH, anti-mullerian hormone; LPS, lipopolysaccharides; TNF, tumor necrosis factor; IL, interleukin.

**Table S3.** Primer sequences for RT-qPCR.

| Gene | Primer | Sequence (5' to 3') |  |
| --- | --- | --- | --- |
| *Gapdh* | Forward | AGGTCGGTGTGAACGGATTTG |  |
|  | Reverse | TGTAGACCATGTAGTTGAGGTCA |  |
| *Star* | Forward | ATGTTCCTCGCTACGTTCAAG |  |
|  | Reverse | CCCAGTGCTCTCCAGTTGAG |  |
| *Cyp11a1* | Forward | AGGTCCTTCAATGAGATCCCTT |  |
|  | Reverse | TCCCTGTAAATGGGGCCATAC |  |
| *Cyp17a1* | Forward | GCCCAAGTCAAAGACACCTAAT |  |
|  | Reverse | GTACCCAGGCGAAGAGAATAGA |  |
| *Hsd3b1* | Forward | TGGACAAAGTATTCCGACCAGA |  |
|  | Reverse | GGCACACTTGCTTGAACACAG |  |
| *Cyp19a1* | Forward | ATGTTCTTGGAAATGCTGAACCC |  |
|  | Reverse | AGGACCTGGTATTGAAGACGAG |  |
| *Hsd17b1* | Forward | ACTTGGCTGTTCGCCTAGC |  |
|  | Reverse | GAGGGCATCCTTGAGTCCTG |  |
| *Hsd17b7* | Forward | ACGGGCCAAAAGGTACATAATC |  |
|  | Reverse | CCAGCATGAGGACTCGGAT |  |
